# Identification of Novel Thymic Epithelial Cell Subsets Whose Differentiation Is Regulated by RANKL and Traf6

**DOI:** 10.1371/journal.pone.0086129

**Published:** 2014-01-21

**Authors:** Nichole M. Danzl, Seihwan Jeong, Yongwon Choi, Konstantina Alexandropoulos

**Affiliations:** 1 Columbia Center for Translational Immunology, Columbia University Medical Center, New York, New York, United States of America; 2 Department of Medicine, Division of Clinical Immunology, The Immunology Institute, Icahn School of Medicine at Mount Sinai, New York, New York, United States of America; 3 Department of Pathology and Laboratory Medicine, University of Pennsylvania, Philadelphia, Pennsylvania, United States of America; Institut Pasteur, France

## Abstract

Thymic epithelial cells (TECs) are critical for the normal development and function of the thymus. Here, we examined the developmental stages of TECs using quantitative assessment of the cortical and medullary markers Keratin 5 and Keratin 8 (K5 and K8) respectively, in normal and gain/loss of function mutant animals. Gain of function mice overexpressed RANKL in T cells, whereas loss of function animals lacked expression of Traf6 in TECs (Traf6ΔTEC). Assessment of K5 and K8 expression in conjunction with other TEC markers in wild type mice identified novel cortical and medullary TEC populations, expressing different combinations of these markers. RANKL overexpression led to expansion of all medullary TECs (mTECs) and enlargement of the thymic medulla. This in turn associated with a block in thymocyte development and loss of CD4^+^CD8^+^, CD4^+^ and CD8^+^ thymocytes. In contrast, Traf6 deletion inhibited the production of most TEC populations including cortical TECs (cTECs), defined by absence of UEA-1 binding and LY51 expression, but had no apparent effect on thymocyte development. These results reveal a large degree of heterogeneity within the TEC compartment and the existence of several populations exhibiting concomitant expression of cortical, medullary and epithelial markers and whose production is regulated by RANKL and Traf6.

## Introduction

Normal development of T cells in the thymus depends on interactions between the T cell receptors (TCRs) of developing thymocytes and peptide antigens presented by cortical and medullary thymic epithelial cells (cTECs and mTECs respectively). Self-antigen presentation on cTECs regulates thymocyte positive selection, while self-antigens expressed on mTECs mediate negative selection of autoreactive T cells [1]. Over the past several years mTECs have emerged as important regulators of T cell tolerance by ectopically expressing a wide range of tissue-specific antigens (TSAs) [2]. T cells expressing TCRs that exhibit high affinity for TSAs are eliminated in the thymus through negative selection, whereas TCRs that bind to TSAs with intermediate affinity are diverted into the regulatory T cell (Treg) pool [3], [4], [5], [6], [7].

The transcriptional regulator Aire controls the expression of a large fraction of TSAs in mTECs [8]. Mutations in the *aire* gene in humans result in the development of autoimmune polyendocrinopathy-candidiasis-ectodermal dystrophy (APECED) syndrome [9], [10], [11], whereas Aire deletion in mice leads to autoantibody production against and inflammatory infiltrates in multiple tissues [8]. In addition to Aire, genetic mutations that affect the development of mTECs have varied effects on mTEC function and autoimmunity. Deletion or mutation of RelB [12], the tumor necrosis factor receptor-associated factor 6 (Traf6) [13], NFκB-inducing kinase (NIK) [14] and the lymphotoxin β receptor (LTβR) [15], leads to defective development of mTECs, reduced or absent medulla and development of autoimmunity [16], [17]. In addition, the receptor activator of NFκB ligand (RANKL) and CD40 ligand (CD40L) expressed on CD4^+^ T cells together with their respective receptors RANK and CD40 expressed on mTECs, were recently shown to regulate development and maintenance of mature mTECs [18], [19]. RANKL and CD40L are selectively upregulated in CD4^+^ T cells and positively selected CD4^+^ thymocytes expressing RANKL are necessary for mTEC cellularity illustrating the role of thymocyte-TEC crosstalk in normal T cell development [17], [20].

Although the development of thymic epithelial cells is not well understood, cTECs and mTECs are thought to arise from a common embryonic progenitor that gives rise to both lineages [21]. In the adult thymus, LY51 and CD205 expression identifies cTECs, while mTECs are LY51^−^CD205^−^ and bind the plant lectin ulex europeus agglutinin-1 (UEA-1). mTECs are further subdivided into three cell subsets expressing different levels of CD80/86, MHCII and Aire [2], [17]. In addition, mTECs express the epithelial cell marker Keratin 5 (K5) whereas cTECs express Keratin 8 (K8). Although K8 and K5 are considered medullary and cortical specific markers, we and others observed heterogeneous expression of both proteins within the thymic medulla, suggesting the existence of TEC populations coexpressing different levels of these proteins [4], [22], [23], [24]. Therefore, we performed quantitative analysis of keratin and other TEC marker expression and used gain and loss of function RANKL and Traf6 mutant mice respectively, to identify additional TEC subsets whose development was regulated by these proteins.

## Materials and Methods

### Animals

Transgenic mouse lines in the C57BL/6 background overexpressing RANKL under the control of the murine CD4 enhancer/promoter lacking the CD4 silencer [25] were described previously [26]. Traf6ΔTEC mice were generated by crossing floxed Traf6 mice [27] to animals in which a cDNA encoding for the Cre recombinase was knocked into the 3′ untranslated region (3′UTR) of the *foxn1* locus [28], [29]. Traf6^fl/fl^/foxn1-Cre (Traf6ΔTEC) mice were backcrossed for 8 generations to the C57BL/6 background. C57BL/6 mice were purchased from Jackson Laboratory (Bar Harbor, ME). Animals were housed in specific pathogen-free conditions and were used and maintained in accordance with institutional guidelines. All efforts were made to minimize suffering. Animal protocols were approved by the Institutional Animal Care and Use Committee of the Icahn School of Medicine at Mount Sinai (Protocol # 09-00102).

### Thymic Epithelial Cell (TEC) Isolation

Individual thymi from different animals were dispersed and single cell suspensions enriched using a percoll gradient as described previously [30], [31]. Briefly, thymi from mice between 7–9 weeks of age were minced into small pieces and enzymatically digested in RPMI1640 containing 0.2 mg/ml Collagenase D (Roche), 6.5 U/ml Dispase I (Roche) and 301 U/µl Dnase I (Invitrogen) at 37°C for 30 min. Upon completion of the digestion, 0.5M EDTA (1∶50 v/v, Invitrogen) was added to the cell suspensions for 5 min and after washing, cells were loaded on a 1.115 g/ml and 1.065 g/ml Percoll (Sigma) density gradient topped off with 1X phosphate buffered saline (PBS). The Percoll/PBS gradient was centrifuged at 2700 rpm for 30 min at 4°C and cell aggregates formed between the PBS and 1.065 g/ml Percoll layer were collected for analysis by flow cytometry.

### Flow Cytometry

Percoll gradient purified TEC suspensions were incubated with anti-CD16/CD32 Fc block (2.4G2, BD Biosciences, 1∶10 dilution) to block Fc receptors along with biotinylated UEA-1 (1∶25, Vector Laboratory) for 25 min at room temperature (RT). Next, the cells were incubated with PerCP-conjugated Streptavidin (1∶50, BD biosciences), anti-MHCII-eFluor450 (1∶50, M5/114.15.2, eBioscience) and −CD45-PE (1∶500, 30-F11, eBioscience) for 25 min at RT. When relevant, anti-EPCAM1-PE-Cy7 (1∶100, G8.8, eBioscience) was added along with the above mentioned antibodies. Affinipure anti-rat Fab fragment (1∶10, Jackson Immunoresearch) was added for 35 min followed by fixation by cytofix/cytoperm solution (BD Biosciences) for 20 min. Fixed cells stained with extracellular markers were resuspended in Perm/Wash buffer (BD Biosciences) overnight, followed by incubation with anti-K8 (TROMA-I clone; DSHB, University of Iowa) and -K5 (AF138 clone; Covance) antibodies 1∶50 dilution for 60 min at RT followed by Perm/Wash buffer wash. The cells were stained with APC-conjugated goat anti-rat IgG (1∶50, Jackson Immunoresearch) and FITC-conjugated goat anti-rabbit IgG (1∶50, Jackson Immunoresearch) for K8 and K5 respectively. Samples were analyzed on an LSRII (BD Bioscience) flow cytometer at the Mount Sinai Flow Cytometer Core Facility and the raw data analyzed with FlowJo software (Treestar). Cells stained with Pacific Blue Rat IgG2b (1∶50, RTK4530, Biolegend), PE-Cy7 Rat IgG2a (1∶100, eBR2a, eBioscience), Streptavidin-PerCP, APC-goat anti-rat IgG and FITC-goat anti-rabbit IgG were used as isotype controls.

For experiments staining for Aire expression, Percoll-gradient-purified TECs were incubated in anti-CD16/CD32 Fc Block and biotylated UEA-1 for 25 min followed by Streptavidin conjugated PerCP, anti-MHCII-eFluor450 and −CD45-PE at the dilutions described above for 25 min followed by fixation/permeabilization solution (eBioscience) for 1 hour. Fixed cells stained with extracellular markers were incubated with anti-Aire-AlexaFluor 647 antibody (1∶100, 5H12, eBioscience) for 1 hour. For experiments staining for cortical marker expression, Percoll-gradient-purified TECs were incubated in anti-CD16/CD32 Fc Block and biotylated UEA-1 for 25 min followed by Streptavidin conjugated PerCP, anti-MHCII-eFluor450, −CD45-PE, -LY51-AlexaFluor 647 (1∶200, 6C3, Biolegend) and -EpCAM1-FITC (1∶100, G8.8, eBioscience) for 25 min. Cells were analyzed by flow cytometry as described above.

### In Vitro Cultures of Sorted TECs

Thymi from 3-week old mice were isolated and pooled TEC suspensions were purified as described above. Percoll gradient purified TEC suspensions were incubated with anti-CD16/CD32 Fc block and biotinylated UEA-1 for 25 min at RT followed by incubation with PerCP-conjugated Streptavidin, anti-MHCII-eFluor450 and −CD45-PE for 25 min at RT. UEA-1^−^MHCII^−^ and UEA-1^lo^MHCII^−^ cell populations were sorted on a BD Influx sorter. Sorted cells were incubated for three days in 96 well plates in DMEM supplemented with 4.5 g/L glucose and L-glutamine and 10% FCS in the presence or absence of stimulating anti-RANK antibody (AF692, R&D Systems, 10 µg/mL) as described [32]. At the end of the incubation, cells were stained with anti-MHCII-eFluor450, −EPCAM-1-PE-Cy7 and -CD45-PE. Stained cells were analyzed by flow cytometry.

### Immunohistochemistry

Frozen thymic sections (7 µm) from adult mice (6–9 weeks old) were fixed with ice cold acetone, permeabilized with RPMI 1640 (Gibco) containing saponin (0.05% w/v, Sigma), glycine (10 mM, Fisher Scientific) and Donkey Serum (5% v/v, Sigma) and blocked with egg white (10% v/v) and BSA (0.05% w/v, Sigma). PBST [1x PBS with Tween 20 (0.05% v/v, Fisher Scientific)] was used as wash buffer. The sections were then incubated with anti-K8 (TROMA-I clone; DSHB, University of Iowa) and anti-K5 antibodies (AF138 clone; Covance) at 1∶300 and 1∶1000 dilution respectively for 1 hour at RT followed by incubation with Alexa Fluor 488 anti-rat IgG (1∶200, Invitrogen) and Cy-5 anti-rabbit IgG (1∶200, Invitrogen) secondary antibodies for visualizing K8 and K5 respectively. The sections were also stained with 2 µg/ml rhodamine conjugated UEA-1 (1∶100, Vector Laboratories) for 1 hour at RT. After overnight incubation with biotin-conjugated MHCII (ER-TR3, Abcam) at 1∶100 dilution, Streptavidin-Alex Fluor 350 (1∶100, Invitrogen) was added for an hour. Images of stained tissues were acquired with an Axioplan 2IE fluorescence microscope (Carl Zeiss) at the Mount Sinai Microscopy core facility.

### Lymphocyte Purification and Analysis

Cell suspensions of crushed thymi from wild type and RANKL-Tg mice, were passed through 40 µm cell strainer and suspended in PBS. 2×10^6^ cells were stained with anti-CD45-AlexaFluor780 (30F11, eBioscience), −CD4-PE-Cy7 (GK1.5, eBiosciences), −CD8-eFluor450 (53–6.7, eBioscience), −CD44-PE (IM7, BD biosciences) and −CD25-APC-Cy7 (PC61, BD biosciences) antibodies and analyzed by flow cytometry.

### Statistical Analysis

Statistical significance was assessed using the two-tailed Student’s t-test using Microsoft Excel2010 software. P values less than 0.05 were considered significant. *p<0.05; **p<0.01: ***p<0.001.

## Results

### The Thymic Medulla Consists of Heterogeneous TEC Subsets

**Figure 1:**
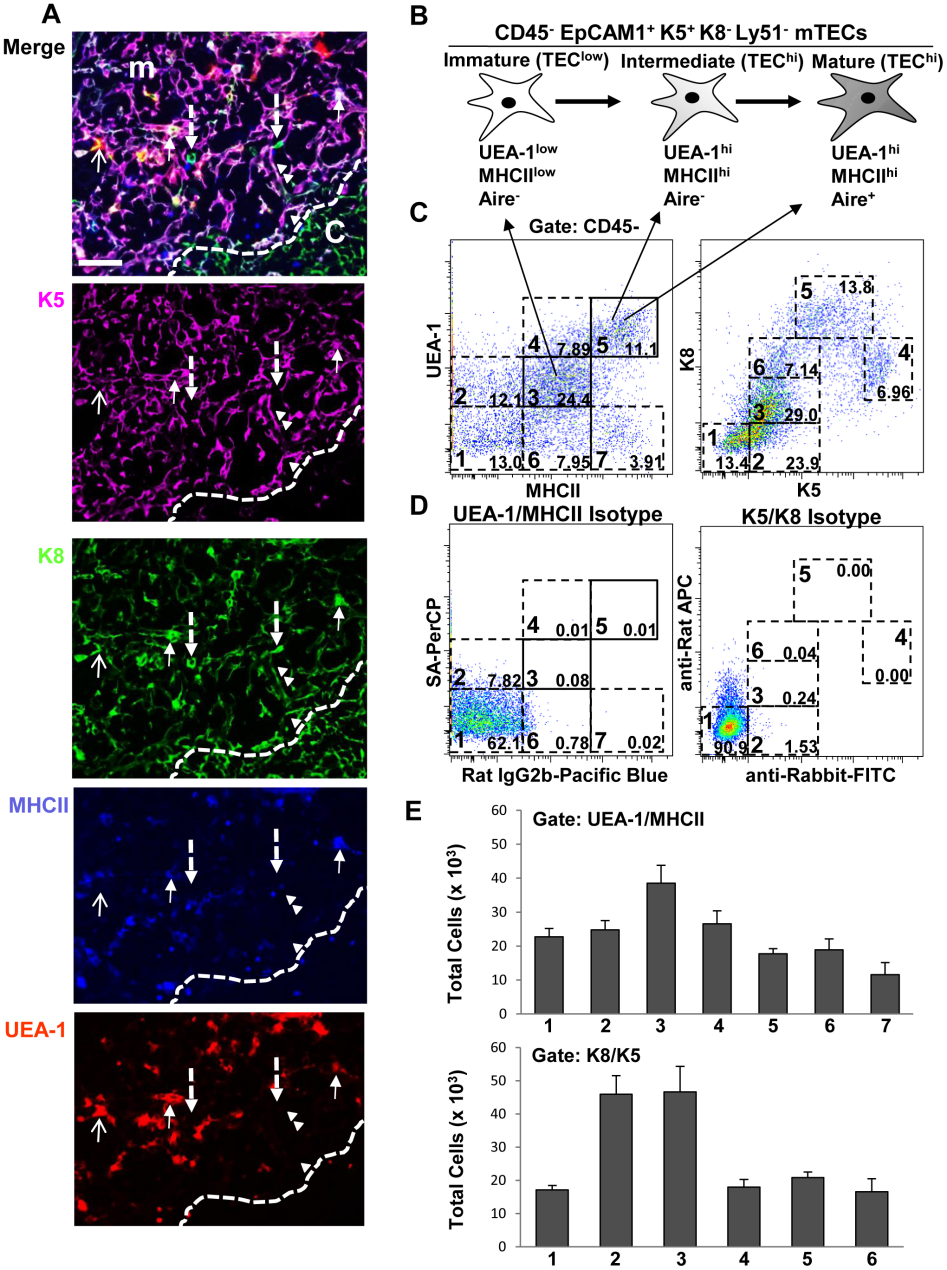
The thymic medulla contains several distinct TEC populations. (**A**) Frozen thymic sections from ∼8-week-old mice were stained with K5, K8, MHCII and UEA-1. Arrows point to different TEC subsets as described in the text. Scale bar = 50 µm. (**B**) Schematic representation of known subsets of mTECs defined by expression/binding levels of MHCII, Aire and UEA-1. (**C**) Purified thymic epithelial cells from ∼8-week-old mice were stained with anti-CD45, -MHCII, -K5 and -K8 antibodies and biotinylated UEA-1, and analyzed by flow cytometry. Seven populations based on UEA-1 binding and MHCII expression (left dot plot) and six populations based on K5 and K8 expression levels were identified (right dot plot). (**D**) Isotype controls for UEA, MHCII, K8 and K5 were included in the experiment as shown. (**E**) The total numbers of the populations within the different gates of UEA/MHCII (1–7) and K8/K5 (1–6) dot plots were quantified by flow cytometry. Bar graphs represent the mean+Standard Error of the Mean (SEM). n = 12, results in C–E were pooled from at least three independent experiments.

Thymic sections of wild type mice were stained with anti-K8, -K5, -MHCII antibodies and UEA-1. While K5 expression was largely confined in the medulla (Figure 1A, top two panels) K8 was present in both the medulla and cortex (Figure 1A, top and third panels). In addition, whereas most K5^+^ cells also coexpressed K8 (Figure 1A, top three panels), some cells in the medulla were positive for only K8 (Figure 1A, top and third panels, dashed arrows). Three populations of mTECs have been defined based on the expression of MHCII, CD80/86, Aire and UEA-1 binding: immature, intermediate and mature [2], [17] (Figure 1B). Immature mTECs express/bind low levels of MHCII/CD80/86 and UEA-1 respectively (TEC^low^), whereas intermediate and mature mTECs express high levels of MHCII/CD80/86, bind high levels of UEA-1 (TEC^hi^) and are Aire^−^ or Aire^+^ respectively (Figure 1B) [2], [17], [33]. TEC^low^ and TEC^hi^ cells were indeed present in the medulla of wild type mice as staining thymic sections with anti-MHCII antibody and UEA-1 revealed cell populations binding low and high levels of these markers (Figure 1A, bottom two panels). Among the K5^+^K8^+^ medullary cells, we identified three distinct populations based on MHCII coexpression and UEA-1 binding: K8^+^K5^+^UEA-1^+^MHCII^+^ (Figure 1A, closed arrows); K8^+^K5^+^UEA-1^+^MHCII^−^ (Figure 1A, open arrow); and K8^+^K5^+^UEA-1-MHCII^−^ cells (Figure 1A, arrowheads). Therefore, the heterogeneous staining of K8, K5, MHCII and UEA-1 suggested the existence of several distinct cell subsets within the medullary compartment.

### Quantitative Assessment of Cortical and Medullary Marker Expression Identifies Novel TEC Subsets

**Figure 2:**
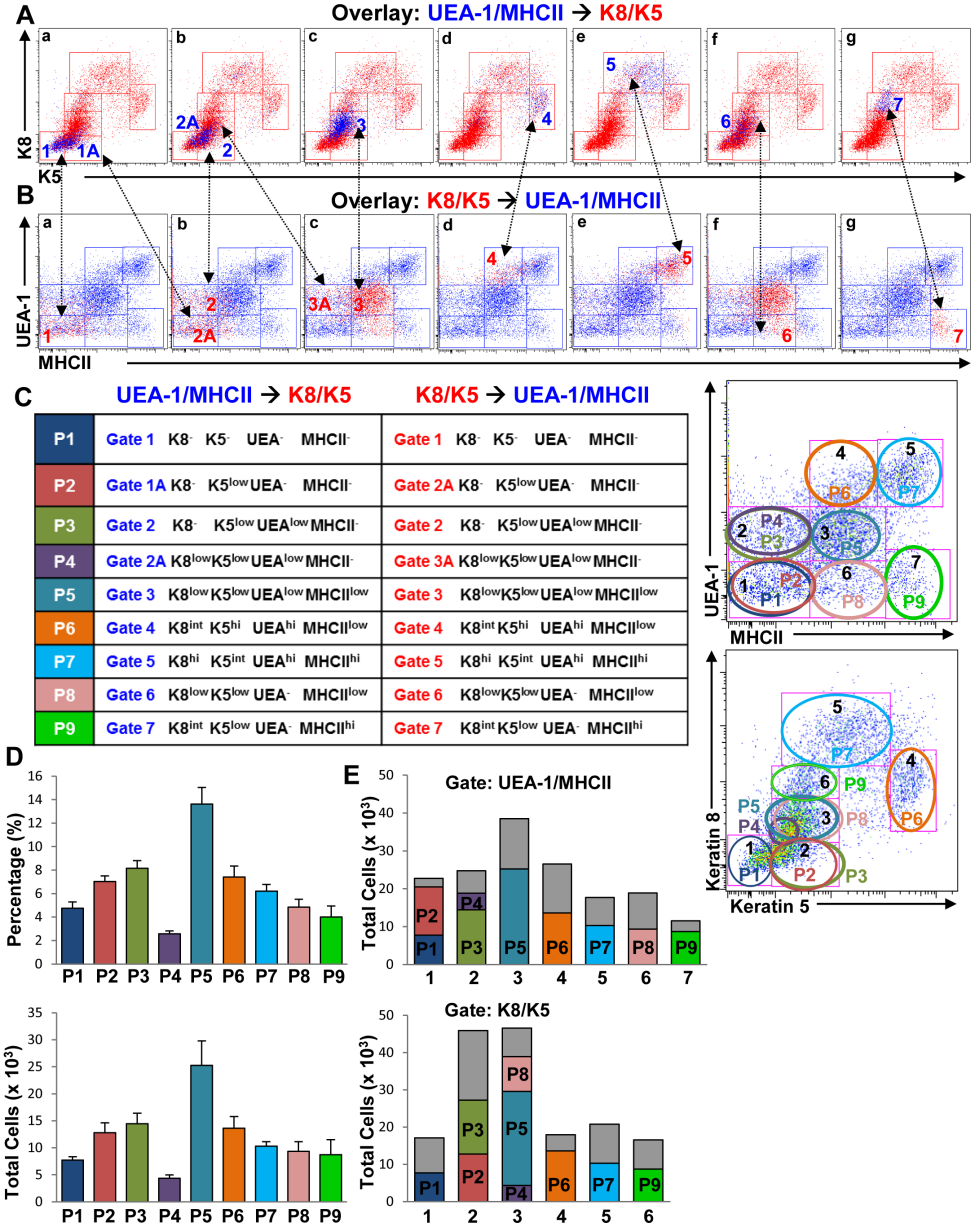
Identification of novel TEC populations by simultaneous expression of cortical and medullary TEC markers. (**A** and **B**) The different cell populations subgated on UEA/MHCII and K8/K5 dot plots from Figure 1C were overlaid onto K5/K8 (A) and UEA/MHCII (B) dot plots respectively (panels **a–g**) to identify cells coexpressing these markers. Panel **f** in B is a replica of **c** to show the overlapping populations within gate 3 of the K8/K5 dot plots. (**C**) Color-coded cell subsets defined in the UEA/MHCII and K8/K5 overlays in A and B were renamed and designated as described in the text and as shown (table). The dot plots show a schematic representation of the overlapping populations defined in A and B. (**D**) Percentages and total numbers of each cell subset were quantified by flow cytometry. (**E**) Bar graphs show the proportion of each cell subset characterized in C (color bars) within the total population of cells gated (gray bars). Bar graphs represent mean+SEM. n = 12 data were pooled from at least three independent experiments.

To further characterize the thymic TEC compartment, we developed a multi-color flow cytometry protocol that allowed simultaneous quantitative analysis of K8, K5, MHCII and UEA-1 expression/binding in TEC subsets. Analysis of TEC suspensions from wild type mice revealed several distinct epithelial (CD45^−^) populations expressing different levels of these markers on UEA/MHCII and K8/K5 dot plots (Figure 1C). The gating strategy for deducing the different populations was determined based on isotype control staining (Figure 1D), marker expression levels and by overlaying subpopulations gated on UEA/MHCII onto K8/K5 dot plots and vise-versa (presented in Figure 2 below). In addition to the known populations pointed by arrows in Figure 1B and C (left panel, solid squares), four other populations were discernible within the UEA/MHCII dot plots of CD45^−^ cells enclosed in dotted squares: UEA-1^low^MHCII^−^; UEA-1^high^MHCII^low^; UEA-1^−^MHCII^low^; and UEA-1^−^MHCII^hi^ (Figure 1C, left panel, gates 2, 4, 6 and 7 respectively). Similarly, six different cell populations expressing variable levels of K8 and K5 were gated on a K8/K5 dot plot based on the same strategy as with the UEA/MHCII plots. The total numbers of the different populations within the UEA/MHCII (gates 1–7) and K8/K5 (gates 1–6) dot plots from several wild type mice were quantified by flow cytometry (Figure 1E).

To further characterize these TEC subpopulations, cells gated on UEA/MHCII dot plots (Figure 1C, left panel) were overlaid onto K8/K5 dot plots (Figure 2A, blue dots) and K8/K5-gated subpopulations (Figure 1C, right panel) overlaid onto UEA/MHCII dot plots (Figure 2B, red dots). Gates 1 of the UEA/MHCII and K8/K5 dot plots (Figure 1C) contained cells that did not express any of the markers tested (Figure 2A and B, panels **a**, populations 1) and were designated K8^−^K5^−^UEA-1^−^MHCII^−^ (Figure 2C, table). The UEA/MHCII gate 1 also contained cells that expressed low levels of K5 and were identical to cells present in gate 2 of K8/K5 dot plots (Figure 2A panel **a**, and 2B panel **b**, populations 1A and 2A). These cells were designated K8^−^K5^low^UEA-1^−^MHCII^−^ TECs (Figure 2C, table). UEA-1 binding was first detected in cells contained within gates 2 of UEA/MHCII and K8/K5 dot plots (Figure 2A and B, **b** panels, populations 2) and these were designated K8^−^K5^low^UEA-1^low^MHCII^−^ TECs (Figure 2C, table). In addition, the UEA/MHCII dot plot gate 2 contained another cell subset in which K8 expression first became discernible (Figure 2A, panel **b**, population 2A). This cell subset overlapped with a population within gate 3 of K8/K5 dot plots (Figure 2B, panel **c**, population 3A) and was designated K8^low^K5^low^UEA-1^low^MHCII^−^ (Figure 2C). Gate 3 on the UEA/MHCII dot plots (Figure 1C, left panel) contained cells that coincided with the previously defined TEC^low^ subset [34], in which MHCII upregulation first became evident (Figure 2A and B, **c** panels, populations 3) and was designated K8^low^K5^low^UEA-1^low^MHCII^low^ TECs (Figure 2C). Further increases in K8 and K5 expression correlated with MHCII upregulation and UEA-1 binding in populations 4 and 5 (Figure 2A and B, panels **d** and **e** respectively) designated K8^int^K5^hi^UEA-1^hi^MHCII^low^ and K8^hi^K5^int^UEA-1^hi^MHCII^hi^ (Figure 2C), the latter representing the previously described (TEC^hi^) mature TEC cell subset (Figure 1B) [33].

In addition to the UEA-1^+^ cell populations mentioned above, we also identified UEA-1^−^ cells in our TEC cell preparations that expressed low and high levels of MHCII (Figure 1C, left panel, populations 6 and 7). Overlaying UEA/MHCII gates 6 and 7 onto K8/K5 dot plots showed that these cells expressed low levels of K5, K8 and MHCII (Figure 2A and B, **f** panels, populations 6) designated as K8^low^K5^low^UEA-1^−^MHCII^low^ (Figure 2C, table). This population was also contained in gate 3 of K8/K5 dot plots and overlapped with the 3 and 3A cell subsets (Figure 2B, compare identical panels **c** and **f**). Cells in gate 7 of UEA-1/MHCII dot plots expressed intermediate and high levels of K8 and MHCII respectively (Figure 2A and B, panels **g** populations 7) and were designated as K8^int^K5^low^UEA-1^−^MHCII^hi^ (Figure 2C, table).

The cell subsets identified in the overlays above were color coded and renamed as populations 1–9 (P1–P9) (Figure 2C, table). The overlapping subpopulations within gates 1 and 2 in the UEA/MHCII dot plots and gates 2 and 3 in the K8/K5 dot plots are shown schematically in Figure 2C (dot plots) and the percentages and total numbers of the overlaid populations determined by flow cytometry are shown in Figure 2D. Because the overlaid populations in Figure 2A and B did not account for all the cells gated in the UEA/MHCII and K8/K5 dot plots shown in Figure 2C (dot plots), the proportion of cells expressing all four markers (Figure 2E, colored bars) was determined in relation to the total populations gated (Figure 2E, gray bars). Although the identity of the cells represented in the gray bars is unknown, these could be other TEC subsets expressing variable combinations of the markers tested. Consistent with this, both K8^+^K5^+^UEA-1^+^MCHII^+^ and K8^+^K5^+^UEA-1^−^MCHII^−^ cells were present in the thymic medulla of wild type animals (Figure 1A, arrows and arrowheads respectively). However, the opposite was not true as we were not able to detect K8^−^K5^−^UEA-1^+^MHCII^+^ cells in any of the thymic sections examined. Together, these results revealed the existence of several TEC subpopulations coexpressing variable levels of the different markers tested, suggesting a greater complexity of the TEC compartment than previously thought.

### Further Characterization of the Identified TEC Subsets Using Epithelial and Cortical Marker Expression

**Figure 3:**
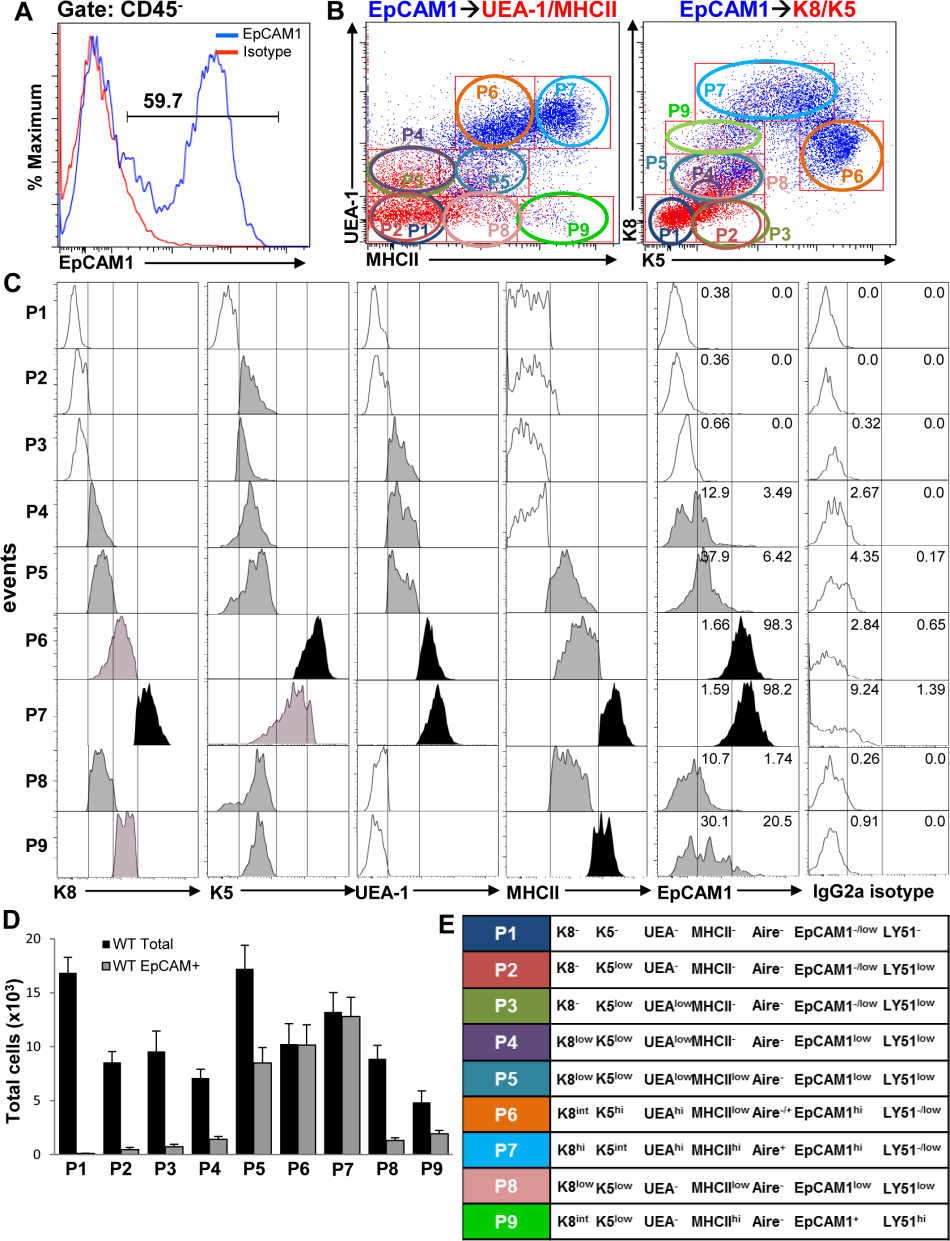
Rare EpCAM1^+^ cells are present in the different TEC cell subsets. (**A** and **B**) EpCAM1^+^ cells gated on CD45^−^ epithelial cells were overlaid on UEA/MHCII and K8/K5 dot plots (blue dots). (**C**) EpCAM1 and other marker expression levels for each cell subset were analyzed by flow cytometry on histograms. An isotype control was used to differentiate EpCAM1^+^ from EpCAM1^−^ cells. (**D**) Total numbers of all CD45^−^ cells gated as well as EpCAM1^+^ cells within the different gates were determined by flow cytometry. (E) Nomenclature assignments of the TEC subsets identified. Bar graphs represent the mean+SEM. n = 16, results were pooled from at least three independent experiments.

In addition to MHCII, K8, K5 and UEA-1, known TEC subsets also express the pan-epithelial cell marker EpCAM1 [17]. Additionally, LY51 expression has been used to distinguish cTECs from mTECs, where LY51^hi^EpCAM1^+^ cells have been shown to represent cTECs and LY51^−/low^EpCAM1^+^ cells have been described as mTECs [17], [31]. EpCAM1 expression on the different cell subsets was analyzed in conjunction to the other markers by overlaying CD45^–^gated EpCAM1^+^ TECs (Figure 3A) onto UEA/MHCII or K8/K5 dot plots as described in Figure 2 and as shown (Figure 3B). Consistent with the results presented in Figure 2C, histogram analysis of individual TEC markers revealed that upregulation of K5 followed by increased UEA-1 binding were the first markers to be detected on TEC subsets (Figure 3C, populations P2 and P3 respectively). EpCAM1^+^ cells were also detected in the P3 and more so in the P4–P5 populations (Figure 3C), whereas almost all cells in the P6 and P7 subpopulations expressed high levels of EpCAM1^+^ which coincided with increased UEA-1 binding and in the case of P7, MHCII expression (Figure 3C and D and Figure S1B–F). The total numbers and frequency of EpCAM1^+^ cells in the different populations contained within the total cells gated were determined by flow cytometry (Figure 3D and Figure S1A). The expression levels of EpCAM1 and the other markers examined were confirmed by mean fluorescence intensity (MFI) (Figure S1B–F) and the designations of the different TEC populations deduced from overlays, histograms and MFI values are summarized in Figure 3E. Based on UEA-1binding and K5 expression we believe that the P3–P7 cell subsets represent mTECs where the P2 subset expressing low levels of K5 may be representative of an early stage along the mTEC lineage differentiation.

In contrast to the P3–P7 cells subsets we defined, the P8 and P9 subpopulations did not bind UEA-1 despite expressing different levels of K5, K8, MHCII and EpCAM1 (Figure 3C and Figure S1B–F), suggesting that these cells may represent cTECs. K8^+^K5^+^ double positive cells that were MHCII^+^UEA-1^−^ were previously observed at the corticomedullary junction (CMJ) of the thymus and were described as minor cortical cells proposed to serve as precursors to both cTECs and mTECs [24]. To further characterize these populations we stained thymic TEC cell suspensions with LY51 and EpCAM1. At least three different populations were discernible expressing high and low levels of these markers determined from LY51/EpCAM1 dot plots and MFI values (Figure S2A and B) and as described [31]. Overlaying LY51^hi^ cTECs (Figure S2A, gate 1) onto UEA-1/MHCII dot plots overlapped with gate 7 containing the P9 subset (defined in Figure 3B), although LY51^hi^ cells that bound low levels of UEA-1 were also present (Figure S2B, left panel and histogram). LY51^−/low^ EpCAM^hi^ mTECs (Figure S2A, gate 2) overlapped with gates 4 and 5 (Figure S2B middle panel) containing populations P6 and P7 (defined in Figure 3B), whereas LY51^low^EpCAM1^low^ cells were distributed between gates 1, 2, 3 and 6 on UEA-1/MHCII dot plots (Figure S2B, right panel) containing the P1–5 and P8 cell subsets (defined in Figure 3). Although cells within gates 6 and 7 containing the P8 and P9 cell subsets respectively lacked the ability to bind UEA-1, they differed in their LY51 expression levels (Figure S2C, histogram). Therefore, while P9 consists at least partially of cTECs, P8 cells appear to represent a distinct TEC population. As these cells express low levels of both K5 and K8 but not UEA-1 (Figure S1C–E) and K8^+^K5^+^UEA-1^−^ cells have been proposed to act as mTEC and cTEC precursors [24], it is possible that the P8 population contains these precursors. The designations for LY51 expression on the different subsets are shown in Figure 3E.

**Figure 4:**
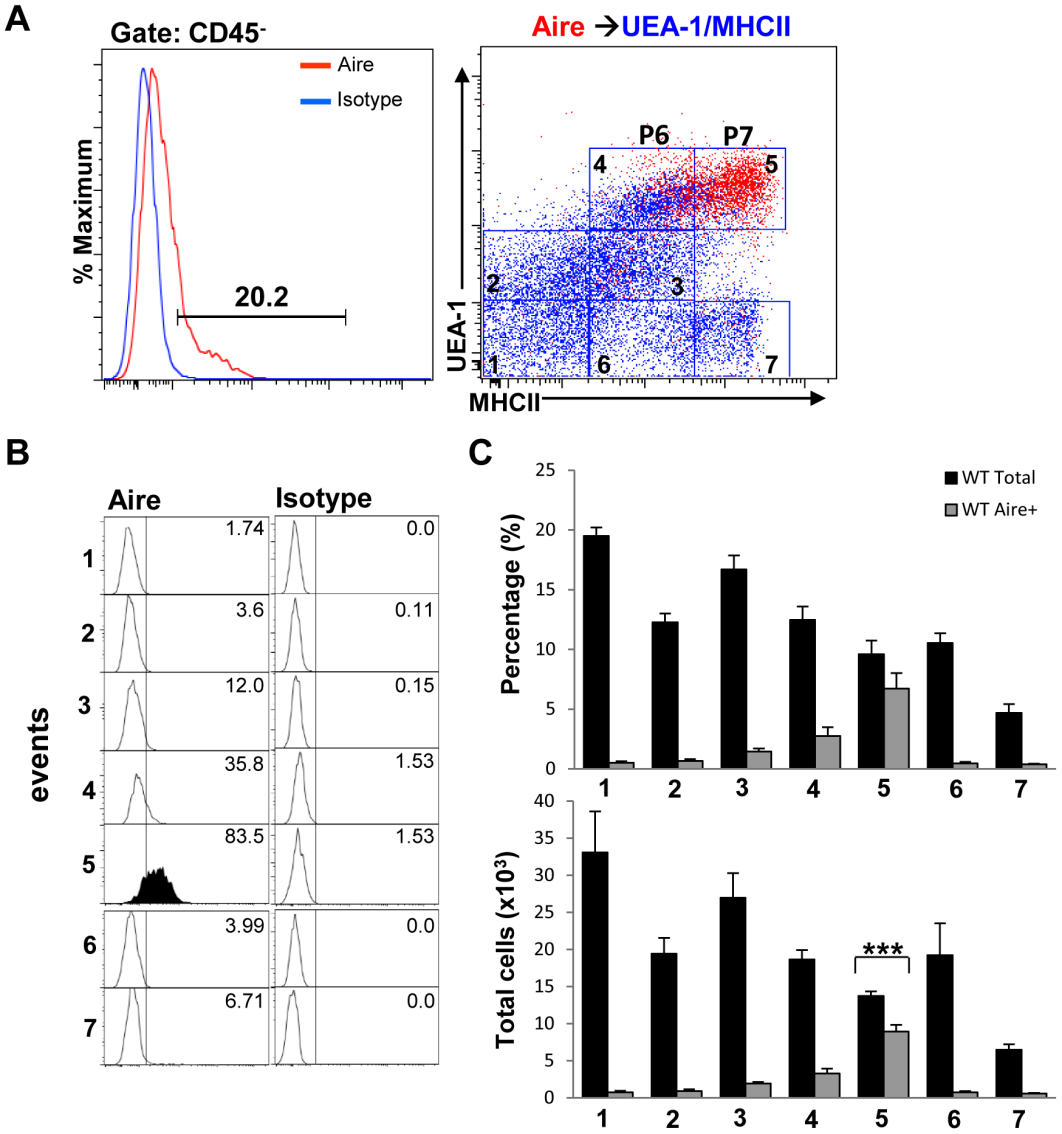
Aire expression is upregulated in the mature TEC^hi^ cell subset. (**A**) Aire-expressing cells within the CD45^−^ gate were overlaid onto UEA-1/MHCII dot plots of TEC suspensions (right panel, red dots). (**B**) Aire expression levels within each cell population gated on the UEA/MHCII dot plot in 4A were analyzed by flow cytometry on histograms. (**C**) The percentages and total numbers of Aire^+^ cells within the different UEA-1/MHCII-gated populations (1–7) were determined by flow cytometry. Bar graphs represent mean+SEM with n = 10 from 3 different experiments. (**D**) Table shows the name designations of the different subsets based on expression levels of all markers tested.

Aire expression and TSA induction have been shown to occur in the most mature UEA-1^hi^MHCII^hi^ mTECs [17], [33]. As a proof of principle and to determine which of the different subsets we identified contained Aire^+^ cells, in parallel experiments we also examined Aire expression in the different mTEC subpopulations we defined above (P2–P7). Because of limited availability of antibody clones and fluorescent conjugates, we were unable to simultaneously assess Aire expression with all of the other markers. Therefore, the percentages and total numbers of Aire^+^ cells were determined in UEA-1/MHCII dot plots. Overlay of Aire-expressing cells within the CD45^−^ gate onto UEA-1/MHCII dot plots, (Figure 4A, red dots), revealed that the majority of Aire^+^ cells were contained within gate 5 Figure 4A and B) corresponding to the P7 cell subset defined in Figure 3. A minority of Aire^+^ cells was also present in gate 4 of the same dot plots corresponding to the P6 population of mTECs (Figure 4A and B). Quantification by flow cytometry of the percentages and total numbers of Aire^+^ mTECs within the different populations gated on UEA/MHCII dot plots are shown in Figure 4C. As not all cells in the P7 subset expressed Aire (Figure 4C, bottom bar graph), these results were consistent with previous evidence showing that the most mature TEC^hi^ cell subset consists of both Aire^−^ and Aire^+^ mTECs [17], [33]. Because cells in the P7 cell subset contained within gate 5 (Figure 4A, right panel, red dots) also express intermediate and high levels of K8 and K5 as well as EpCAM1 (Figure 2A, **d** and **e** panels and Figure 3C), we believe that these cells can be designated as K8^hi^K5^int^UEA-1^hi^MHCII^hi^Aire^+^EpCAM1^hi^ representing the previously described TEC^hi^ Aire^+^ mature mTECs. The phenotypes of the cell subsets we characterized with respect to K8, K5, MHCII, Aire and EpCAM1 expression and UEA-1 binding, are summarized in Figure 3E. Together, these results suggest that K8 expression is not solely confined in the thymic cortex but rather coexpression of cortical, medullary and epithelial markers is dynamically regulated in different TEC subsets in the thymic medulla.

### RANKL and Traf6 Regulate the Expansion of the Identified EpCAM1^+^ TEC Subsets

**Figure 5:**
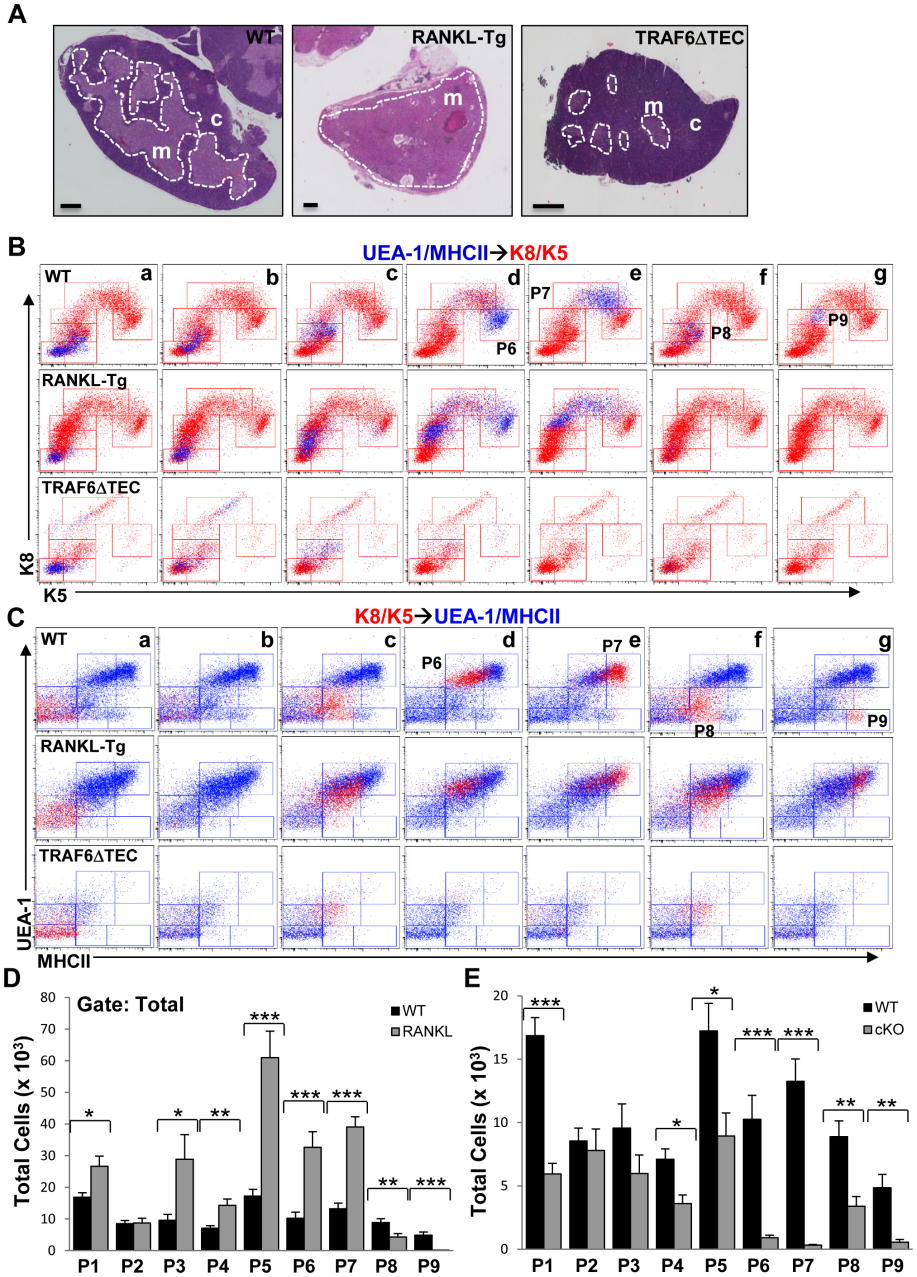
RANKL and Traf6 regulate the expansion of TEC subsets. (**A**) Mosaic images of H&E sections of thymic lobes from ∼3-month-old RANKL-Tg and Traf6ΔTEC mice show the effect of RANKL expression and Traf6 deletion on medullary architecture. Scale bars = 500 µm**.** (**B** and **C**) UEA/MHCII→K8/K5 (blue dots) and K8/K5→UEA/MHCII (red dots) overlays generated as in Figure 2A and B comparing patterns of UEA-1, MHCII, K8 and K5 expression in wild type, RANKL-Tg and Traf6ΔTEC mice. (**D** and **E**) Quantification of the total numbers of TEC subpopulations in wild type and RANKL-Tg (D) or Traf6ΔTEC (cKO) mice (E) within the total gated populations as shown in Figure 1C. Bar graphs represent mean+SEM. At least 9 mice were used per genotype from four independent experiments.

As RANKL and Traf6 have been shown to regulate TEC differentiation [19], [35], we used gain and loss of function mutations for these proteins to examine their impact on the populations we identified. RANKL transgenic (RANKL-Tg) mice overexpressing membrane-bound RANKL in T cells and Traf6ΔTEC conditional knockout mice were generated as described in Materials and Methods. Transgenic RANKL expression was detected on CD4^+^CD8^+^ double positive (DP), and CD4^+^ and CD8^+^ single positive (SP) thymocytes by flow cytometry (data not shown). Hematoxilin and eosin (H&E)-stained cross-sections of thymic lobes from ∼12-week old wild type and RANKL-Tg mice revealed a marked expansion of the thymic medulla in RANKL-Tg animals which associated with greatly reduced or absent cortex (Figure 5A, left and middle panels). In contrast, deletion of Traf6 in TECs resulted in a diminished medullary area (Figure 5A, right panel), consistent with previously published results with straight Traf6 knockout and conditional Traf6ΔTEC mice [13], [29].

UEA/MHCII→K8/K5 and K8/K5→UEA/MHCII overlays performed as in Figure 2A and B, revealed several qualitative and quantitative differences in TEC subsets between wild type and RANKL-Tg or Traf6ΔTEC mice (Figure 5B and C). Overexpression of RANKL skewed TEC populations towards the P6 and P7 mTEC subsets in terms of UEA-1 and MHCII expression compared to wild type controls (Figure 5C middle panels and D). The P6 and P7 cell subsets in the UEA/MHCII→K8/K5 overlay of TECs from RANKL-Tg mice exhibited a downward shift in K5 expression levels compared to wild type controls, which correlated with a concomitant increase in immature populations expressing lower levels of K5 (Figure 5B, compare top and middle **d** and **e** panels). The P6 and P7 cell subsets bound and expressed similar levels of UEA-1 but there was an increase in cells expressing lower levels of MHCII within the P6 population on the K8/K5→UEA/MHCII overlay of RANKL-Tg mice (Figure 5C, compare top and middle **d** and **e** panels). Despite the differences in expression levels of K5 and MHCII in the P6 and P7 cell subpopulations, RANKL expression led to the expansion of most of the P1–P7 cell subsets suggesting that these cells indeed represent mTECs (Figure 5D). This was also consistent with the ability of these cells to bind UEA-1 and with the role of RANKL in mTEC regulation. In contrast, the P8 and P9 populations were reduced or absent from the UEA-1/MHCII→K8/K5 and K8/K5→UEA/MHCII overlays of RANKL-Tg TECs compared to controls (Figure 5B and C, compare top and middle **f** and **g** panels, and Figure 5D). The decrease in populations P8 and P9 coincided with the depletion of the cortex in the RANKL-Tg thymi (Figure 5A) which together with their inability to bind UEA-1 is consistent with the idea that these cells comprise minor cTECs. The cells represented by red dots in the same RANKL-Tg dot plots (Figure 5C, middle **f** and **g** panels) likely represent mature mTECs expressing lower levels of K5 (shown in Figure 5B, top an middle **d** and **e** panels). In contrast to RANKL overexpression, Traf6 deletion in TECs resulted in severe depletion of the P6 and P7 subsets evident in both the UEA/MHCII→K8/K5 and K8/K5→UEA/MHCII overlays compared to controls (Figure 5B and C, top and bottom **d** and **e** panels and 5E). Deletion of Traf6 had a less potent effect on the P2–P5 populations as these cell subsets were still present in the overlays (Figure 5B and C top and bottom **a–c** panels) and their total numbers were similar to or reduced as compared to controls (Figure 5E). The P8 and P9 populations were also reduced or depleted by Traf6 deletion compared to controls, suggesting that in addition to mTECs Traf6 also regulates the production of minor cortical cells (Figure 5B and C, top and bottom **f** and **g** panels, and 5E).

**Figure 6:**
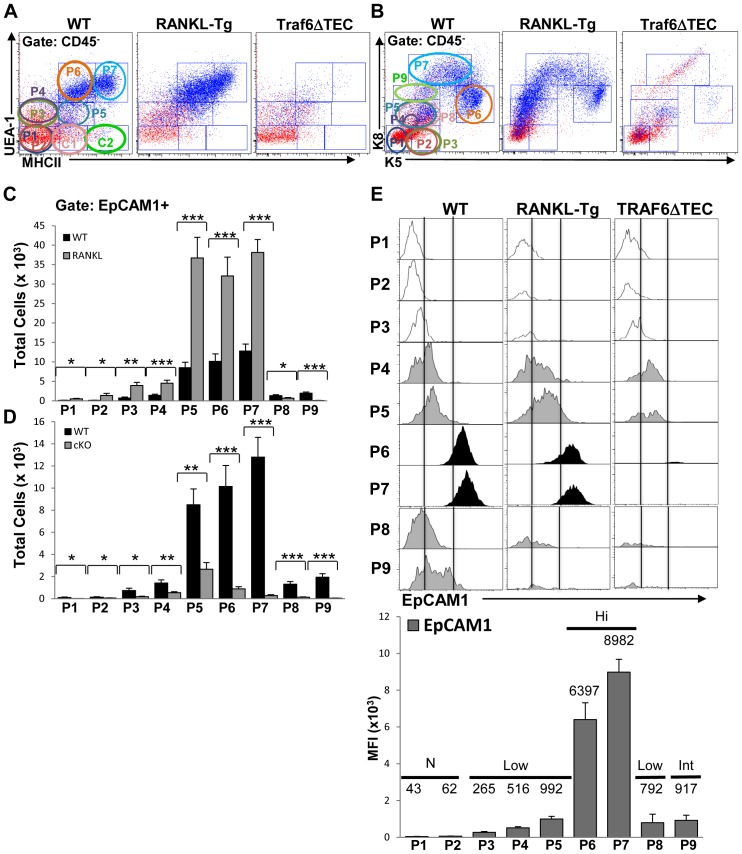
RANKL overexpression and Traf6 deletion regulate the expansion of EpCAM1^+^ TECs. (**A** and **B**) CD45^–^gated EpCAM1^+^ cells from ∼8-week-old mice were overlaid onto UEA/MHCII and K8/K5 dot plots as in Figure 3 and as shown (blue dots). (**C** and **D**) The total numbers of EpCAM1^+^ TECs in the different subsets gated on UEA/MHCII and K8/K5 dot plots (circles in A and B) from wild type, RANKL-Tg and Traf6ΔTEC mice were analyzed by flow cytometry. Bar graphs represent the mean+SEM. Results were pooled from four independent experiments, at least nine mice were used for each genotype. (**E**) EpCAM1 expression for each cell subset in wild type, RANK-Tg and Traf6ΔTEC mice were analyzed by flow cytometry on histograms. MFI values for EpCAM1 expression levels on the different TEC subsets (P1–P9) from RANKL-Tg mice were determined by flow cytometry. Levels of expression were determined as N (no expression), Low, Int (intermediate) and Hi (high) expression.

CD45^−^EpCAM1^+^ cells in the different cell subsets from wild type and RANKL-Tg or Traf6ΔTEC mice were also analyzed on EpCAM1→UEA/MHCII and EpCAM1→K8/K5 overlays as described in Figure 3 above and as shown (Figure 6A and B, blue dots). RANKL overexpression caused significant increases in all except the P8 and P9 EpCAM1^+^ cell subsets which were significantly reduced in RANKL-Tg mice. There was a large increase in the EpCAM1^+^ cell numbers in the P5 cell subset which showed a 6- and 8-fold increase in wild type and RANKL-Tg mice respectively compared to the P4 subpopulation (Figure 6C). These results were consistent with previous evidence showing that TEC^low^ mTECs (corresponding to our P5 cell subset) are comprised of immature proliferating cells [17], [33]. Despite the expansion of the P5 cell subset, cells within this population expressed low levels of EpCAM1 in both wild type and RAKL-Tg mice (Figure S1B and 6E, MFI values), while EpCAM1 levels increased in the P6 and P7 cell subsets without further expansion of these cells (Figure 6C and E and S1B). These results suggest that the P4 and P5 subpopulations may represent a transitional checkpoint in TEC differentiation that involves expansion of cells expressing low levels of EpCAM1 followed by EpCAM1 upregulation without further expansion in the P6 and P7 subsets. In contrast to RANKL-Tg mice, EpCAM1^+^ cell subpopulations isolated from Traf6ΔTEC animals were significantly decreased compared to controls with the most dramatic inhibition evident in the P5 and especially the P6 and P7 cell subsets (Figure 6D), which contrasted with RANKL-mediated expansion of these populations (compare Figure 6C and D). Both RANKL overexpression and Traf6 deletion induced significant changes in the P1 cell which could be due to the existence of rare EpCAM1^+^ cells within this population. EpCAM1^+^ cells within the P8 and P9 subsets were decreased by both RANKL overexpression and Traf6 deletion (Figure 6C and D), which in the case of RANKL-Tg mice was consistent with the absence of a cortical region in their thymus (Figure 5A, middle panel). Together, the results described above suggest that the TEC compartment exhibits a complex differentiation program involving several subpopulations whose expansion and marker upregulation in controlled by RANKL and Traf6.

### Temporal Regulation of TEC Subset Expansion in the Postnatal Thymus

Given the different levels of EpCAM1 and MHCII on the different TEC subsets and to explore a possible precursor role for the P1–P4 populations expressing no or low levels of MHCII and EpCAM1 respectively, we sorted cells within gates 1 and 2 on UEA-1/MHCII dot plots containing these populations (Figure 2C dot plots) and as shown in Figure S3A and incubated sorted cells with a stimulating anti-RANK antibody in vitro (Figure S3). The rational for these experiments was to examine whether RANK-mediated stimulation of sorted populations was able to give rise to MHCII^+^ cells in vitro which would then suggest that the sorted populations contained precursors to more mature mTECs. Staining cultured cells with EpCAM1 and MHCII revealed that the percentages of EpCAM1^+^ TECs in anti-RANK-stimulated cultures were significantly increased compared to untreated cells (Figure S3B). This was accompanied by a modest increase in the levels of EpCAM1 (Figure S3C, left histogram) mirroring the results we obtained with RANKL-Tg mice (Figure 6C and E) however, these in vitro cultures failed to yield MHCII^+^ TECs (Figure S3C, right histogram). These results suggest that although the P1–P4 cell subsets contain cells that respond to RANK stimulation, upregulate EpCAM1, and may act as precursors of more mature TECs additional events are required for upregulation of MHCII and TEC maturation. This would then be consistent with the existence of a checkpoint between populations P4 and P5 mentioned above regulating a transitional expansion and/or survival of cells within the P5 cell subset.

**Figure 7:**
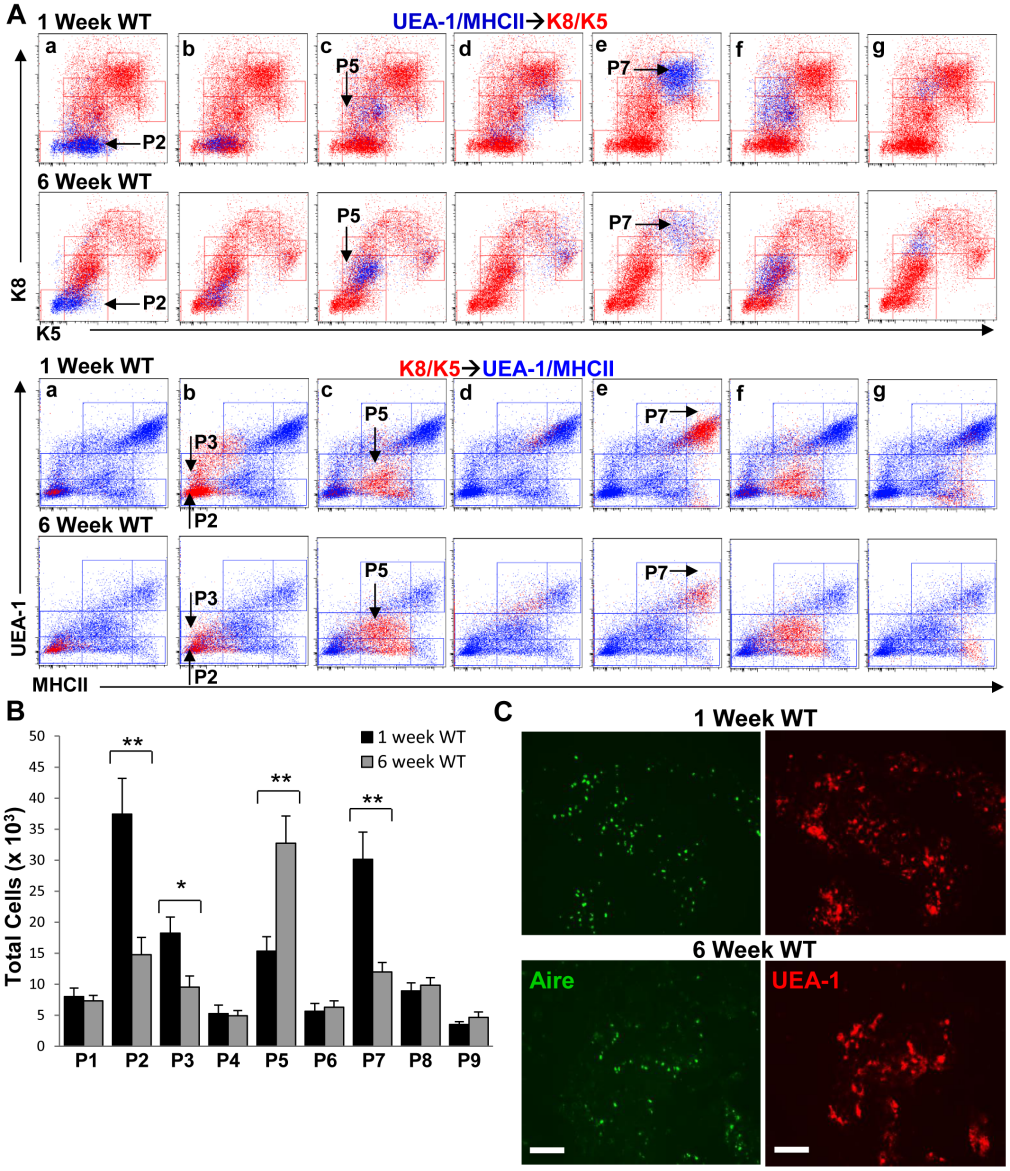
Different TEC subsets are temporally regulated during postnatal thymus development in wild type mice. (**A**) UEA/MHCII→K8/K5 (blue dots) and K8/K5→UEA/MHCII (red dots) overlays were generated as in Figure 2A and B comparing patterns of UEA-1, MHCII, K8 and K5 expression in 1-week-old vs. 6-week-old wild type mice as shown. (**B**) Quantification of the total numbers of TEC subpopulations in 1-week old and 6-week-old wild type mice within the total gated populations was performed by flow cytometry. Bar graphs represent mean+SEM with n = 9 from three independent experiments. (**C**) Frozen thymic sections from 1- and 6-week-old wild type were stained with anti-Aire FITC and rhodamine-conjugated UEA-1 and analyzed by fluorescence microscopy. Scale bar = 100 µm.

**Figure 8:**
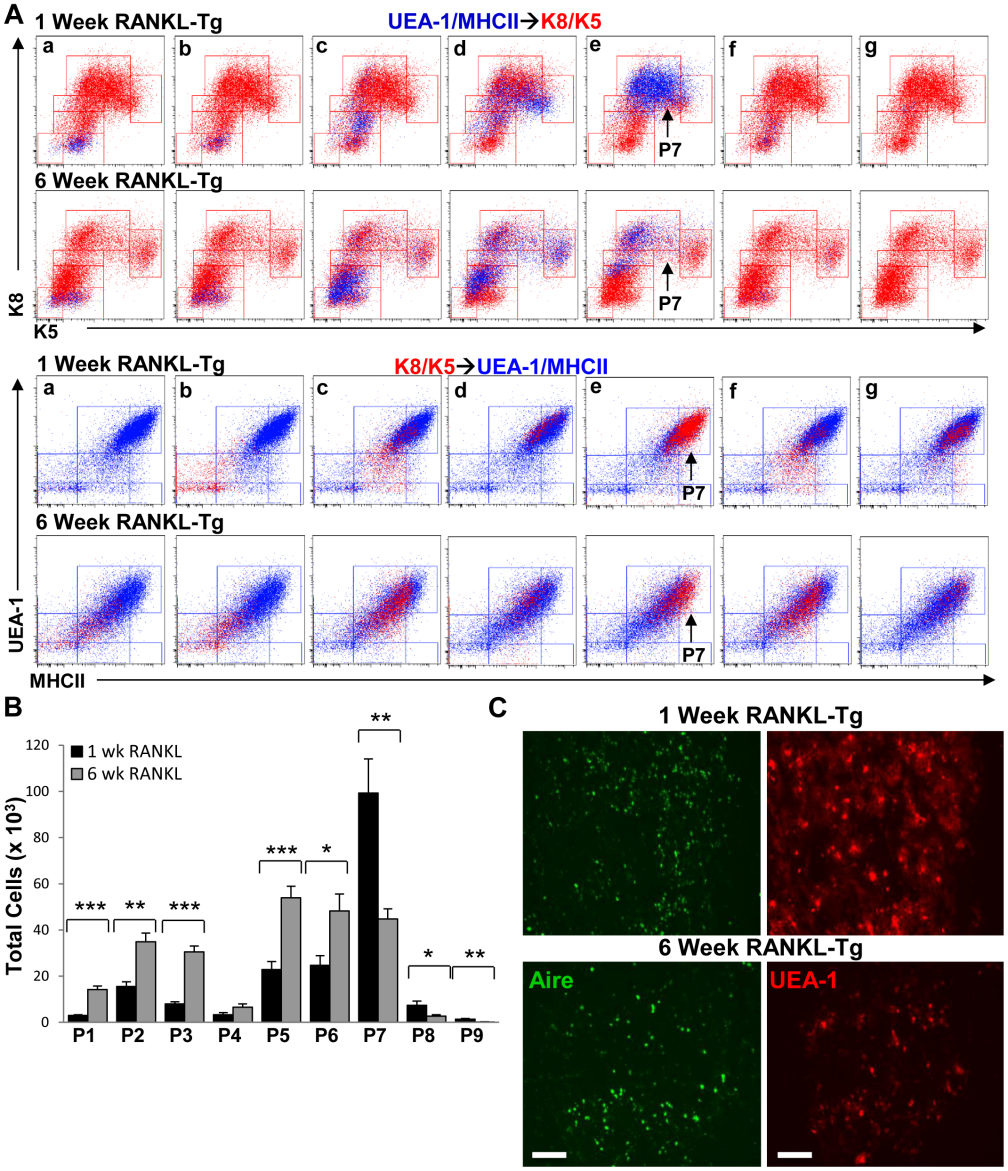
RANKL differentially regulates TEC expansion in the post-natal vs. mature thymus. (**A**) UEA/MHCII→K8/K5 (blue dots) and K8/K5→UEA/MHCII (red dots) overlays generated as in Figure 2A and B comparing patterns of UEA-1, MHCII, K8 and K5 expression in 1- vs. 6-week-old RANKL-Tg mice. (**B**) Quantification of the total numbers of TEC subpopulations in 1- and 6-week-old RANKL-Tg type mice was performed by flow cytometry. Bar graphs represent mean+SEM with n = 9 from three independent experiments. (**C**) Frozen thymic sections from 1- and 6-week-old RANKL-Tg mice were stained with anti-Aire FITC and rhodamine-conjugated UEA-1 and analyzed by fluorescence microscopy. Scale bar = 100 µm.

To gain additional insight into the role of the subsets we identified in TEC development, we analyzed TEC cell suspensions in 1- and 6-week-old wild type and RANKL-Tg mice. We chose these time points because we and others have shown that small medullary islets in newborn mice expand and fuse to form a cohesive medulla within a week after birth and which reaches is normal size by six weeks of age [31], [36], [37]. Based on the perinatal expansion of the medulla, we reasoned that there should be more precursor cells to facilitate medullary formation and production of mature mTECs. UEA-1/MHCII→K8K5 and K8/K5→UEA-1/MHCII overlays revealed significant increases in the P2 and P3 cell subsets in 1-week-old compared to 6-week-old wild type mice whereas the P5 subset was enriched in 6-week old animals consistent with the results shown above in Figure 5D (Figure 7A and B). The opposite was true in 6-week-old RANKL-Tg mice in that all populations except the P8 and P9 subsets were increased compared to 1-week old animals (Figure 8A and B), suggesting that RANKL-RANK signaling may be differentially required at different stages of postnatal thymus development. Interestingly, there was a pronounced enrichment of the P7 subset in 1-week-old wild type and RANKL-Tg animals representing Aire^+^ mTECs (as defined in Figures 3E and 4 above) (Figure 7 and 8 A and B), suggesting that different TEC subsets are differentially abundant at different stages of postnatal thymus development. The increase in the P7 subset (Aire^+^ mTECs) in 1-week-old wild type and RANKL-Tg mice was corroborated by an increase in the presence of UEA-1^+^Aire^+^ mTECs in thymic sections of 1- vs. 6-week-old mice (Figure 7 and 8 C). Collectively, these results suggest the existence of temporal regulation of TEC subsets in the postnatal thymus. The P2 and P3 populations could serve as progenitors whose expansion is necessary to accommodate the expanding neonatal thymus and facilitate the production of Aire^+^ mTECs for the purpose of tolerizing the emerging T cell repertoire. Consistent with this, it was previously shown that Aire was essential during a perinatal window to induce long-lasting T cell tolerance and prevent multiorgan autoimmunity [38].

### Immature TEC Precursors are Present in the Adult Thymus

To further corroborate our results described above, we also performed 4-color immunohistochemistry on thymic sections. Consistent with the results that RANKL overexpression led to expansion of mTECs, we found increased staining with MHCII, UEA-1 and Aire in the thymi of RANKL-Tg mice compared to wild type controls (Figure S4A and B, bottom two panels and data not shown). Expression of K5 and K8 was present throughout the thymic sections and there was no demarcation between the cortical and medullary compartments (Figure S4B). The fact that all of the K5^+^ cells in RANKL-Tg thymi were also K8^+^ supports our observations that K8 expression is widely expressed in mTEC subsets. Moreover, as we found no or very few cortical (K8^+^K5^−^) cells in thymic sections from RANKL-Tg mice (Figure S4B and Figure 5A), our results suggest that medullary expansion in RANKL-Tg mice occurs at the expense of the cortex.

In contrast to RANKL-Tg mice, staining of thymic sections from Traf6ΔTEC mice revealed a medulla devoid of UEA-1^hi^MHCII^hi^ mature mTECs (P7 subset) consistent with the marked inhibition of this population in the knockout mice (Figure S4C, bottom two panels and Figure 5E). TEC subsets coexpressing K5 and K8 were still evident within the medulla and corticomedullary junction (CMJ) of Traf6ΔTEC mice (Figure S4C, top panel, solid and dashed arrows respectively) whereas K8^+^ cells were present in the thymic cortex of these mice (Figure S4C, 1^st^ and 3^rd^ panels). The K8^+^K5^+^ subsets within the medulla but not the CMJ also bound low levels of UEA-1 (Figure S4C, 2^nd^, 3^rd^ and bottom panels), and expressed low levels of MHCII (Figure S4C, second panel from bottom). The K8^+^K5^+^UEA-1^low^ cells present in the medulla of Traf6ΔTEC mice may represent the P4 and P5 (K8^low^K5^low^UEA-1^low^MHCII^−^ and K8^low^K5^low^UEA-1^low^MHCII^low^ respectively) mTEC subsets (Figure 3E), which were still present in the thymus of these mice albeit at reduced numbers than in wild type animals (Figure 5E). The presence of the P4 and P5 TEC populations in the thymi of Traf6ΔTEC mice suggests that in addition to Traf6 other factors may also regulate the development of these mTEC subsets whereas Traf6 is absolutely required for the production of the P6 and P7 populations. In addition to K8^+^K5^+^UEA-1^+^ cells present in the thymic medulla of Traf6ΔTEC mice, K8^+^K5^+^UEA-1^−^ cells were also evident in thymic CMJ of these animals (Figure S4C, dashed arrows) as well as the CMJ of wild type mice (Figure S5). We believe that these cells are the previously characterized minor cortical K8^+^K5^+^ CMJ cells mentioned above represented by the P8 subset (Figure S4C, bottom two panels). Thus, the immunohistological and flow cytometry data presented recapitulate known aspects of TEC development and further delineate the profiles of additional novel TEC subsets.

### RANKL-mediated Medullary Expansion Inhibits T Cell Development

**Figure 9:**
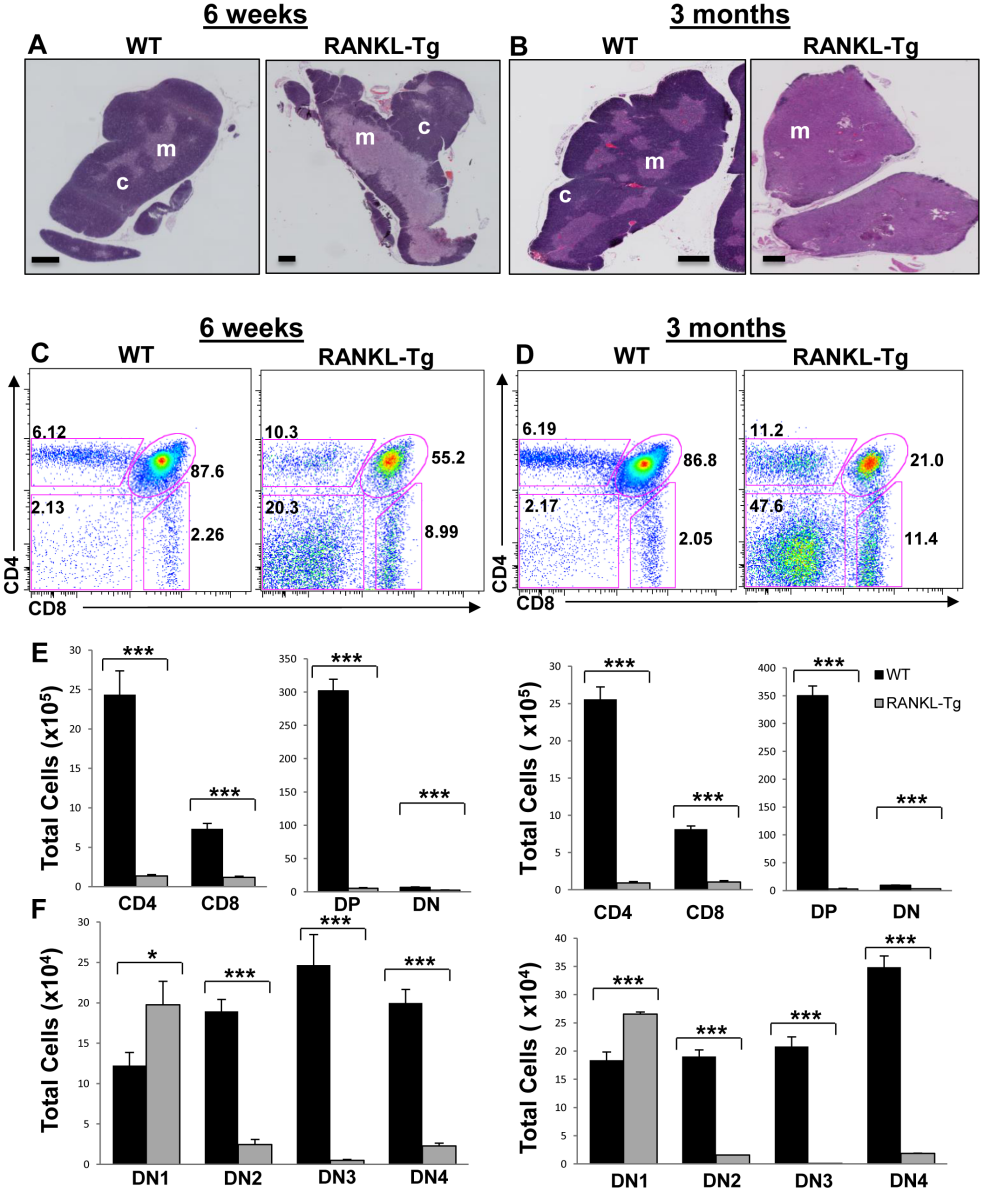
RANKL-mediated medullary expansion inhibits T cell development. (**A and B**) Mosaic images of H&E stained thymic sections of 6-week and 3-month old wild type and RANKL-Tg mice showing progressive expansion of the medulla in response to RANKL expression. Scale bar = 500 µm. Micrographs are representative of at least three independent experiments. (**C–F**). Thymocyte suspensions from 6-week and 3-month old wild type and RANKL transgenic mice were stained with anti-CD4, -CD8, -CD44 and-CD25 antibodies and the total numbers of the different thymocyte subpopulations were analyzed by flow cytometry. Bar graphs represent the mean+SEM**.** n = 9 for each genotype, results were pooled from at least three independent experiments. DP = double positive; DN1-4 = double negative stages 1–4.

We finally examined the effect of the expansion and reduction of the thymic medulla in RANKL-Tg and Traf6ΔTEC mice respectively on T cell development. This process was apparently normal in Traf6ΔTEC mice as similar percentages and total numbers of CD4^+^CD8^+^ double positive (DP) and CD4^+^ and CD8^+^ single positive (SP) cells were present in the thymi, as well as in the spleen and lymph nodes of these animals [29]. However, consistent with the role of mTECs in the elimination of autoreactive T cells, these animals developed peripheral organ-restricted autoimmunity primarily affecting their liver [29]. In contrast, RANKL overexpression led to a marked expansion of the medulla 6 weeks after birth and was further exacerbated at 3 months of age (Figure 9A and B). This medullary expansion in RANKL-Tg animals correlated with altered thymocyte development evident as decreased percentages of CD4^+^CD8^+^ double positive (DP), increased percentages of CD4^+^, CD8^+^ thymocytes, and skewed CD4:CD8 cell ratios towards the CD8 lineage (Figure 9C and D and data not shown). Despite the increased frequency of the different thymocyte populations, the total numbers of all thymocyte subsets were markedly reduced in RANKL-Tg mice compared to control animals (Figure 9E).

The decrease in DP thymocytes in RANKL-Tg mice was accompanied by a concomitant increase in the frequency of CD4^−^CD8^−^ double negative (DN) cells compared to control mice (Figure 9C and D right dot plots and data not shown). Staining of thymic cell suspensions with anti-CD25 and -CD44 antibodies revealed significant increases in the total numbers of the DN1 (CD44^+^CD25^−^) thymocyte population in RANKL-Tg mice compared to controls suggestive of a block in T cell development (Figure 9C, D and F). This increase was not due to the presence of non-T cell lineage cells as exclusion of B cells, monocytes/macrophages and NKT1.1 with specific antibodies had no effect on the DN1 population of RANKL-Tg mice (data not shown). The increase in the DN1 population associated with a marked reduction in the total cell numbers of thymocytes in DN2–DN4 differentiation stages in 6-week and 3-month-old RANKL-Tg mice compared to wild type controls (Figure 9C, D and F). The block in thymocyte development and reduction in total thymocyte numbers associated with peripheral lymphopenia, as the numbers of T cells in the spleen and lymph nodes of RANKL transgenic animals were drastically reduced compared to controls (data not shown). These results suggest that medullary expansion and reduced cortical area due to RANKL overexpression in thymocytes leads to a block in thymocyte development. We believe the lack of sufficient cortical mass may be the cause of defective thymocyte development as antigen presentation by cTECs in the cortex is required for survival, expansion and positive selection of thymocytes [3], [39], [40].

## Discussion

Existing evidence suggests that Aire upregulation correlates with a rapid turnover of mature TECs, suggesting that there is continued replenishment of this cellular compartment from a pool of progenitor cells in the thymus [41]. Despite this evidence, it is unclear whether the CD80/86^low^MHCII^low^ cell subset exclusively gives rise to mature CD80/86^hi^MHCII^hi^Aire^+^ mTECs or whether other unidentified mTEC progenitors exist [42], [43]. Because the thymic medulla exhibited cellular heterogeneity, in this report we assessed keratin expression in relation to other epithelial markers to identify novel TEC populations. A total of nine populations were identified which were classified as mTECs and minor cTECs based on UEA-1 binding and/or expression of the cortical marker LY51 and K8/K5. The earliest detectible expression of any of the markers tested was that of K5 in the P2 subset followed by low levels of UEA-1 binding in the P3 population. Contrary to the notion that K8 is a cTEC marker, K8 expression was first expressed in the UEA-1^+^ P4 subpopulation, and steadily increased in the P5–P7 cell subsets with the highest expression levels evident in the P7 population. Upregulation of MHCII expression occurred in the P5 subset which represents the previously characterized TEC^low^ (UEA-1^low^CD80^low^MHCII^low^) population [17], [33]. In addition, we found another MHCII^low^ population binding high levels of UEA-1 (P6) that we believe represents an intermediate novel population between the TEC^low^ (P5) and TEC^hi^ (P7) mTEC subsets. However, the possibility that these cells represent the involucrin^+^ end-stage maturation mTECs characterized by loss of Aire expression and downregulation of CD80 and MHCII cannot be excluded [35], [44], [45]. K8 expression was highest and coincided with upregulation of Aire expression in the P7 subset representing the previously characterized TEC^hi^ population [17], [33], whereas K5 expression was downregulated to intermediate levels in the same cells. Whether K5 downregulation in the most mature Aire^+^ mTECs is of functional significance is currently unclear. Together, these results suggest that K8 and K5 expression is not solely confined in the thymic cortex or medulla respectively but rather expression of these proteins together with MHCII upregulation and UEA-1 binding is dynamically regulated in different mTEC and cTEC subsets. The different populations may represent different stages of TEC differentiation where the P2 subset expressing low levels of K5 may be representative of an early stage along the mTEC lineage differentiation followed by cells in stages P3–P4 whereas the P5 and P7 subsets represent the known TEC^low^ and TEC^hi^ mTECs respectively.

As CD80/86 and MHCII upregulation was previously linked to mTEC maturation [33], [41], the progressive upregulation of cortical, medullary and epithelial markers observed within the P2–P9 cell subsets, could be indicative of a precursor-product relationship between the different populations. Support for such precursor-product relationship was provided by the experiments using the RANKL-Tg and Traf6ΔTEC mice. RANKL expression significantly increased while Traf6 deletion reduced the total numbers of the TEC subsets particularly cells within the CD45^−^EpCAM1^+^ subsets (P2–P7). The low numbers of EpCAM1^+^ cells within the P2–P4 populations corroborate the existence of a small number of precursor cells that may give rise to increasingly larger numbers of progeny as they proceed to maturity. Support for the idea that the P2–P3 TEC subsets may act as mTEC precursors was also provided by the observation that these populations were enriched in the thymus of 1- vs. 6-week-old wild type mice and that these populations contained EpCAM1^+^ cells that responded to RANK stimulation in RANKL-Tg mice and in in vitro cultures. It is possible that enrichment of these populations in the young thymus is required for the accompanying increased production of mature cells coinciding with the window of Aire-mediated tolerance of the emerging T cell repertoire [38]. Our attempts to directly show that the P2–P3 subpopulations act as precursors to more mature lineages expressing MHCII, while showing the existence of RANK-responsive cells within these populations, failed to produce MHCII^+^ TECs suggesting that other events may be required for progression of these cells to more mature lineages. Our efforts were also hindered by the phenotypic overlap of these cell subsets which prevented sorting of individual populations. Therefore, future in vivo experiments with sorted populations using additional surface markers will be required to definitively establish a precursor product relationship between the different populations we identified.

**Figure 10:**
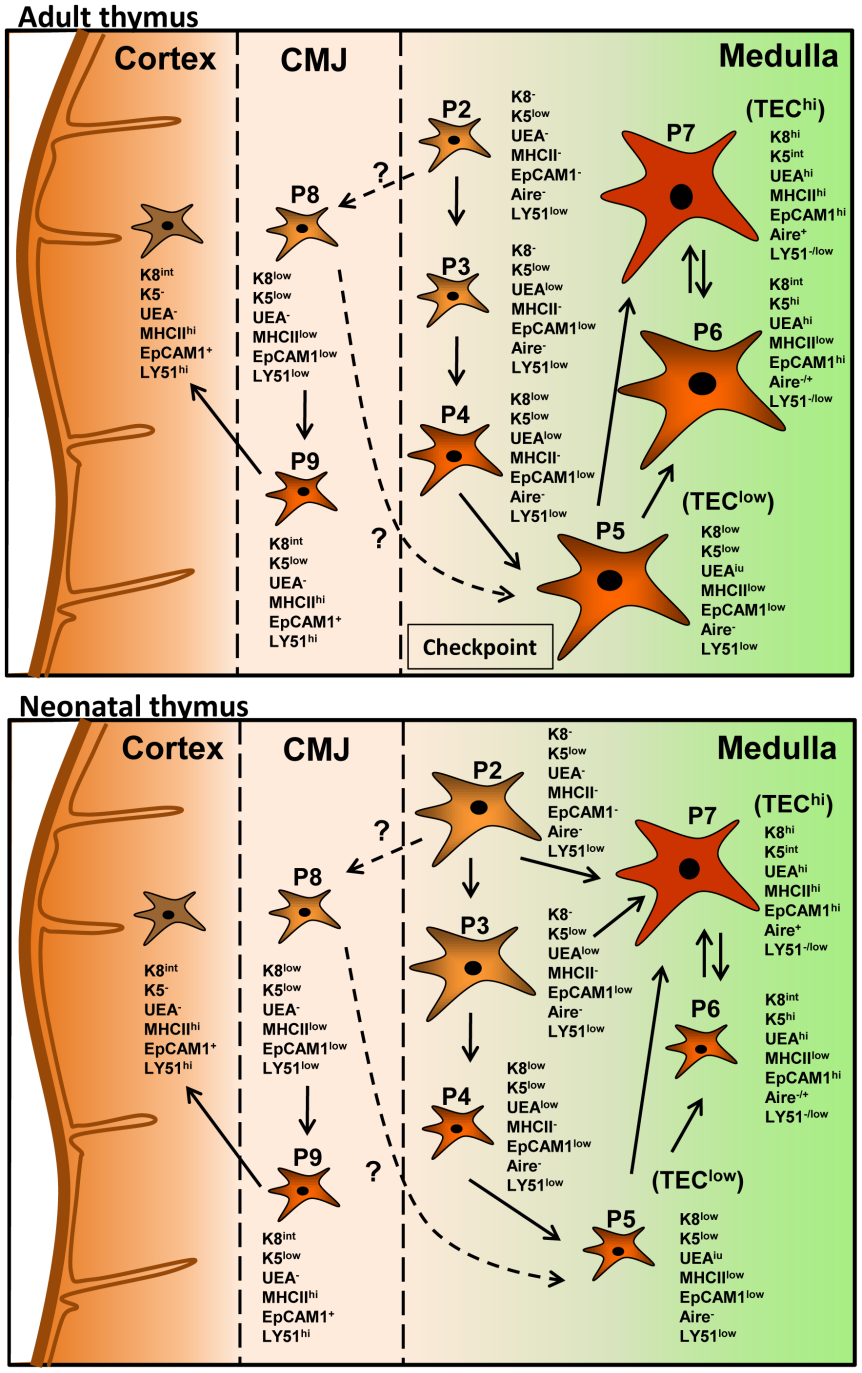
Model of thymic epithelial cell development. Production of TEC subsets is temporally regulated in the adult vs. the neonatal thymus. In the adult thymus the P2 cell pool contains cells expressing low levels of K5 which may serve as precursors to both the P3 and P8 populations. Expression of the UEA-1 receptor in the P3 population commits the cells into the mTEC lineage. The P8 population could serve as a bipotent precursor by upregulating the UEA-1 receptor and contributing to the formation of the P5 subset or by driving the cells into the cTEC lineage through an intermediate P9 population that involves K5 downregulation (as it occurs in the most mature mTECs) and upregulation of MHCII and EpCAM1. The large expansion of the P5 population compared to the P4 subset may represent a checkpoint during TEC differentiation after which P5 cells give rise to mature P7 mTECs either directly or indirectly through the P6 subset. Alternatively the P6 subset may represent terminally differentiated post Aire-stage mTEC expressing low levels of MHCII. In the neonatal thymus, the expanded P2 and P3 cell subsets could give rise to the mature P7 population through the same pathway as the adult thymus or directly to promote rapid production of Aire^+^ mTECs (P7) to tolerize the emerging T cell repertoire. Whereas RANKL does not directly regulate the production of CMJ cTECs, Traf6 regulates generation of mTECs either directly or indirectly through CMJ cTECs. Because Traf6 deletion does not interfere with cortex development, other mechanisms contribute to mature cTEC production.

K8^+^K5^+^ cells at the CMJ of the thymus were previously postulated to be TEC precursors and were designated as minor cortical cells based on their lack of binding UEA-1 [24], [46], [47]. We also identified two cell subsets by flow cytometry, P8 and P9 which coexpressed different levels of K8, K5 and MHCII and in the case of P9 high levels of LY51, but bound no UEA-1. K8^+^K5^+^UEA-1^−^MHCII^low^ cells were also shown by immunohistochemistry to occupy the CMJ of the thymus of wild type mice which we believe represent the previously described minor cortical cells. Currently, we cannot exclude the possibility that the K8^+^K5^+^ cells detected by immunohistochemistry in the CMJ are a mix of both the P8 and P9 populations, as it is difficult to ascertain the precise expression levels of proteins by immunostaining. Interestingly, both RANKL overexpression and deletion of Traf6 in TECs significantly inhibited the P8 and P9 cell subsets. The reduction in the P8 and P9 populations in the case of RANKL overexpression could be due to active inhibition of differentiation of these cells by RANKL, or RANKL-mediated preferential shunting of an early precursor (P2) towards the medullary lineage. This would be consistent with the medullary expansion evident in the thymi of RANKL-Tg mice accompanied by a concomitant reduction of the cortex. Similarly, Traf6 may regulate the production of the P8 subset from an EpCAM1^+^ precursor within the P2 population by selective upregulation of K8 expression. The coexpression and up/down regulation of cortical and medullary markers on the different TEC subsets is consistent with recent evidence showing that the cortical marker β5t is expressed at some point during mTEC development and that progenitors expressing CD205 (a cortical marker) give rise to both cTECs and mTECs [32], [48]. Differential expression of surface markers at different stages of postnatal thymus development could also correlate with temporal regulation/expansion of different set subsets in wild type mice. A working model of TEC differentiation based on our findings is schematically presented in Figure 10.

Finally, expansion of the thymic medulla as a result of RANKL expression had a negative impact on T cell development manifested as an early developmental block in thymocyte development. This developmental block associated with reduced total numbers of DP and mature CD4^+^ and CD8^+^ SP thymocytes and peripheral lymphopenia (unpublished results). Our results overall have important implications in human disease conditions because genetic defects that impair proper TEC development or function, as well as disruption of TEC-thymocyte crosstalk have deleterious effects on thymic function. For example, patients with benign medullary epithelial thymomas experience deregulation of lymphocyte positive and negative selection leading to abnormal thymocyte development and proliferation, autoimmunity, and/or immunodeficiency [49], [50]. Therefore, improved understanding of TEC development and the molecular events that regulate their differentiation can lead to therapeutic interventions for managing and/or treating autoimmune diseases and immune deficiencies as well as thymopoiesis during hematopoietic stem cell transplantation.

## Supporting Information

Figure S1 **Different TEC subsets express variable levels of medullary and cortical markers determined by MFI values.** (**A**) The percentages of as EpCAM1^+^ cells within the total CD45^−^ cells gated were quantified by flow cytometry. (**B–F**) Expression levels of each marker on the different cells subsets (P1– P9) was based on mean fluorescence intensity (MFI) values determined by flow cytometry and were assigned to one of four levels of expression as described in Figure 6. Bar graphs represent the mean+SEM. n = 16, results were pooled from at least three independent experiments.
(TIF)

Figure S2 **The P9 cell subset contains cTECs expressing high levels of the cortical marker LY51.** (**A**) TEC cell suspensions from 6 week old mice were stained with anti-CD45, -LY51, -EpCAM1 and -MHCII antibodies and UEA-1 and stained cells were analyzed by flow cytometry on a dot plot. cTECs and mTECs ate shown within gates 1 and 2 respectively where gate 3 represents LY51^low^EpCAM1^low^ cells. (**B**) MFI values of EpCAM1 and Ly51 expression were determined by flow cytometry and levels of expression were assigned as shown. (**C**) The different cell populations (1–3) subgated on LY51/EpCAM1 dot plots from (A) were overlaid onto UEA-1/MHCII dot plots to identify cells coexpressing these markers. LY51 marker expression levels within gates 1–7 were analyzed by flow cytometry on histograms. Bar graphs represent the mean+SEM. n = 3, results shown were representative of three independent experiments.
(TIF)

Figure S3 **In vitro stimulation of P1–P4 cells with anti-RANKL antibody leads to expansion of EpCAM^+^ TECs.** (**A**) TEC suspensions were stained with anti-CD45, -MHCII and UEA-1 and cells were sorted based on negative and low UEA-1 binding and negative MHCII expression as shown (rectangle). Six 3-week old mice were pooled together for sorting. (**B**) Sorted cells were incubated in vitro with anti-RANK antibody and the percentage of EpCAM1^+^ and MHCII^+^ thymic epithelial cells were quantified after 3 days in culture by flow cytometry. (**C**) Expression of EpCAM1 and MHCII on shorted TECs treated with anti-RANK antibody or left untreated for three days in vitro were analyzed on histograms. Bar graphs represent the mean+SEM. n = 3, results were pooled from three independent experiments *p<0.05.
(TIF)

Figure S4 **Immature TECs are present in the thymus of Traf6ΔTEC animals.** (**A–C**) Frozen thymic sections from ∼6–8-week old wild type, RANKL-Tg and Traf6ΔTEC were stained with anti-K5, -K8 and -MHCII antibodies and rhodamine-conjugated UEA-1 and analyzed by fluorescence microscopy. K8^low^K5^low^UEA-1^low^MHCII^low^ mTECs (solid arrows) and K8^low^K5^low^UEA-1^−^MHCII^low^ minor cTECs (dotted arrows) are present in the thymus of Traf6ΔTEC cKO mice whereas the medulla is devoid of UEA^hi^MHCII^hi^ mature mTECs. Micrographs shown are representative of at least three separate experiments. Scale bar = 100 µm.
(TIF)

Figure S5 **The P8 population is present in the CMJ of the wild type thymus.** Frozen thymic sections from ∼6–8-week old wild type mice were stained with anti-K5, -K8, -MHCII antibodies and UEA-1 and analyzed by fluorescence microscopy. Solid and dashed lines demarcate the cortico-medullary junction (CMJ) of the thymus. Arrowheads point to cells that do not bind UEA-1 but express low levels of K5, K8 and MHCII likely representing the P8 population characterized by flow cytometry in Figure 2. Scale bar = 50 µm.
(TIF)
