# Correction: Identification of Novel Thymic Epithelial Cell Subsets Whose Differentiation Is Regulated by RANKL and Traf6

**DOI:** 10.1371/journal.pone.0110921

**Published:** 2014-10-08

**Authors:** The PLOS ONE Staff

Figure 3 and its legend are incorrect. Please see the corrected Figure 3 here.

**Figure 3:**
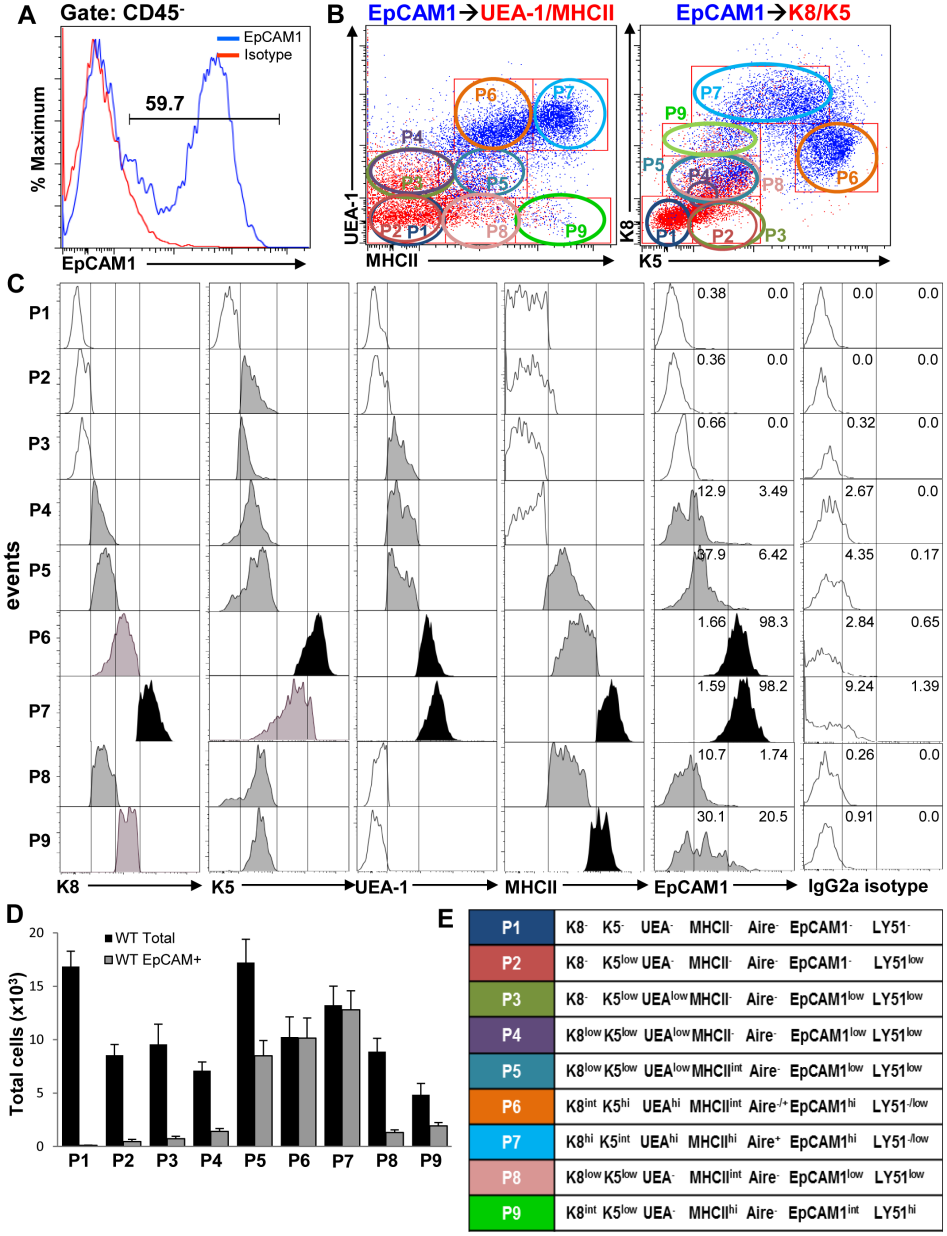
EpCAM1^+^ cell distribution in different TEC cell subsets. (**A** and **B**) EpCAM1+ cells gated on CD45− epithelial cells were overlaid on UEA/MHCII and K8/K5 dot plots (blue dots). (**C**) EpCAM1 and other marker expression levels for each cell subset were analyzed by flow cytometry on histograms. An isotype control was used to differentiate EpCAM1+ from EpCAM1− cells. (**D**) Total numbers of all CD45− cells gated as well as EpCAM1+ cells within the different gates were determined by flow cytometry. (**E**) Nomenclature assignments of the TEC subsets identified. Bar graphs represent the mean+SEM. n  =  16, results were pooled from at least three independent experiments.
