# Supplementary figures and images for: Identification of Novel Thymic Epithelial Cell Subsets Whose Differentiation Is Regulated by RANKL and Traf6
Source: PLoS One. 2014 Jan 21;9(1):e86129. doi: 10.1371/journal.pone.0086129 (PMC3897650; doi:10.1371/journal.pone.0086129)

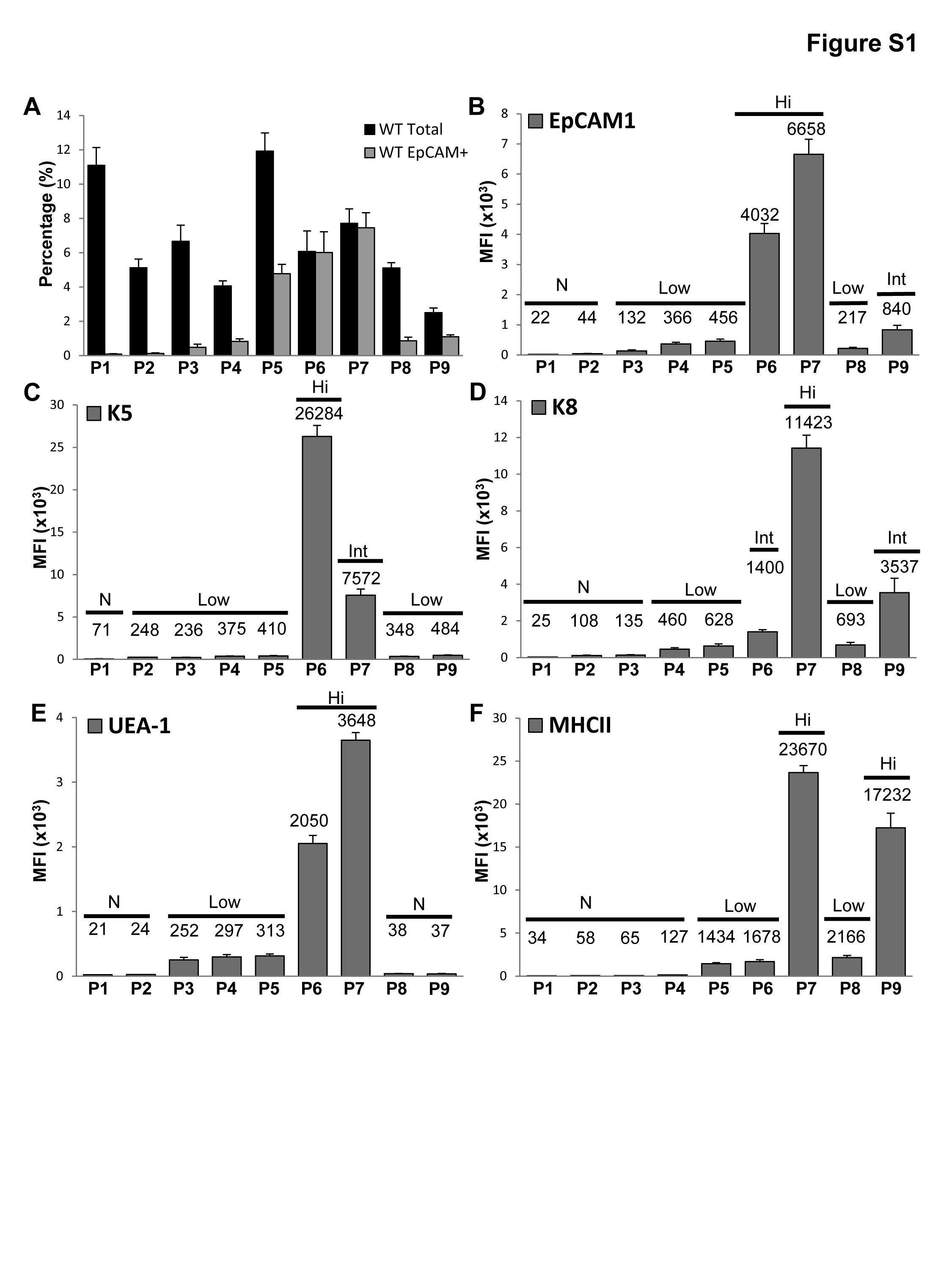

Supplement: Figure S1 — Different TEC subsets express variable levels of medullary and cortical markers determined by MFI values. (A) The percentages of as EpCAM1+ cells within the total CD45− cells gated were quantified by flow cytometry. (B–F) Expression levels of each marker on the different cells subsets (P1– P9) was based on mean fluorescence intensity (MFI) values determined by flow cytometry and were assigned to one of four levels of expression as described in Figure 6. Bar graphs represent the mean+SEM. n = 16, results were pooled from at least three independent experiments. (TIF) [file pone.0086129.s001.tif]

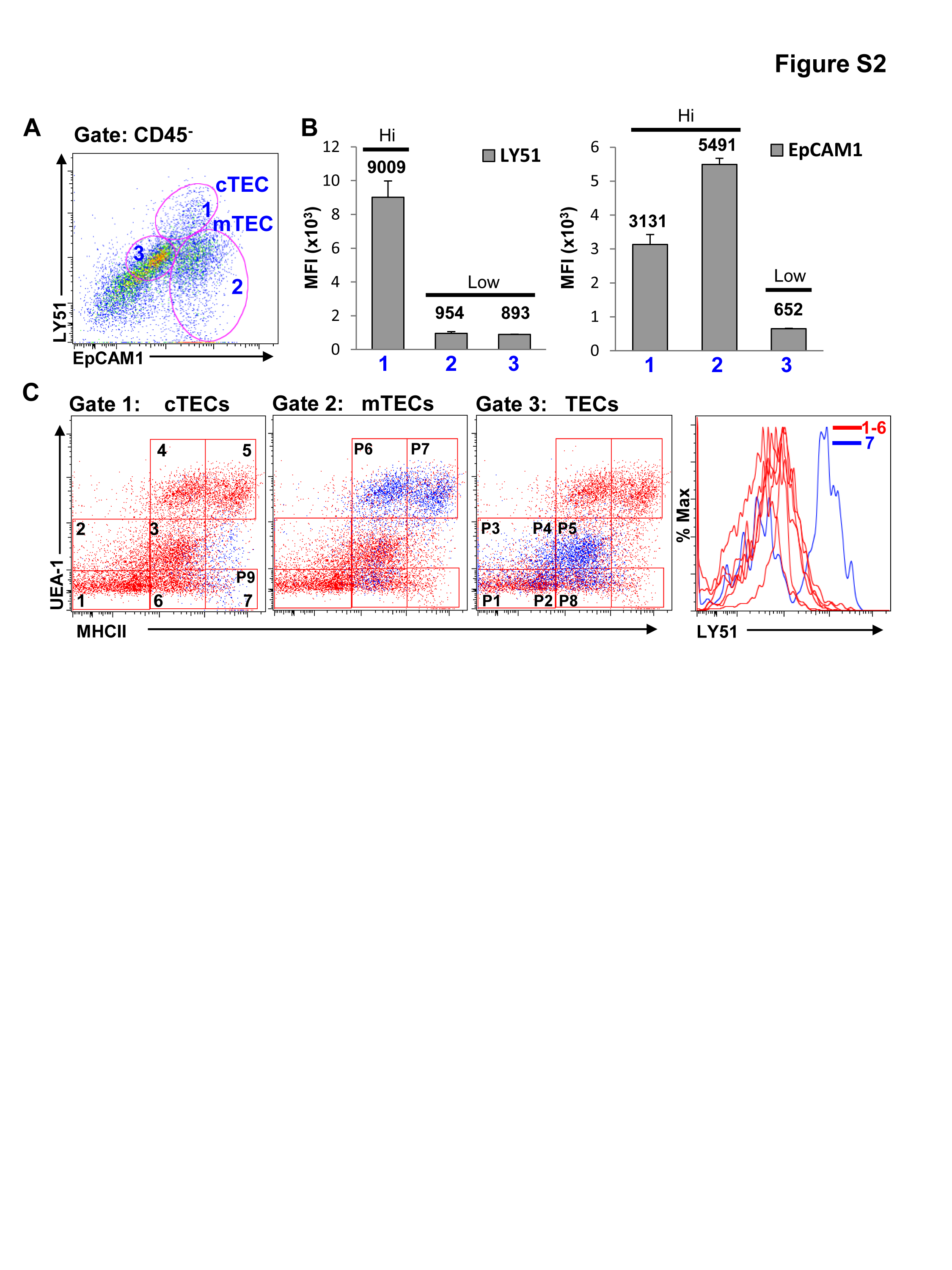

Supplement: Figure S2 — The P9 cell subset contains cTECs expressing high levels of the cortical marker LY51. (A) TEC cell suspensions from 6 week old mice were stained with anti-CD45, -LY51, -EpCAM1 and -MHCII antibodies and UEA-1 and stained cells were analyzed by flow cytometry on a dot plot. cTECs and mTECs ate shown within gates 1 and 2 respectively where gate 3 represents LY51lowEpCAM1low cells. (B) MFI values of EpCAM1 and Ly51 expression were determined by flow cytometry and levels of expression were assigned as shown. (C) The different cell populations (1–3) subgated on LY51/EpCAM1 dot plots from (A) were overlaid onto UEA-1/MHCII dot plots to identify cells coexpressing these markers. LY51 marker expression levels within gates 1–7 were analyzed by flow cytometry on histograms. Bar graphs represent the mean+SEM. n = 3, results shown were representative of three independent experiments. (TIF) [file pone.0086129.s002.tif]

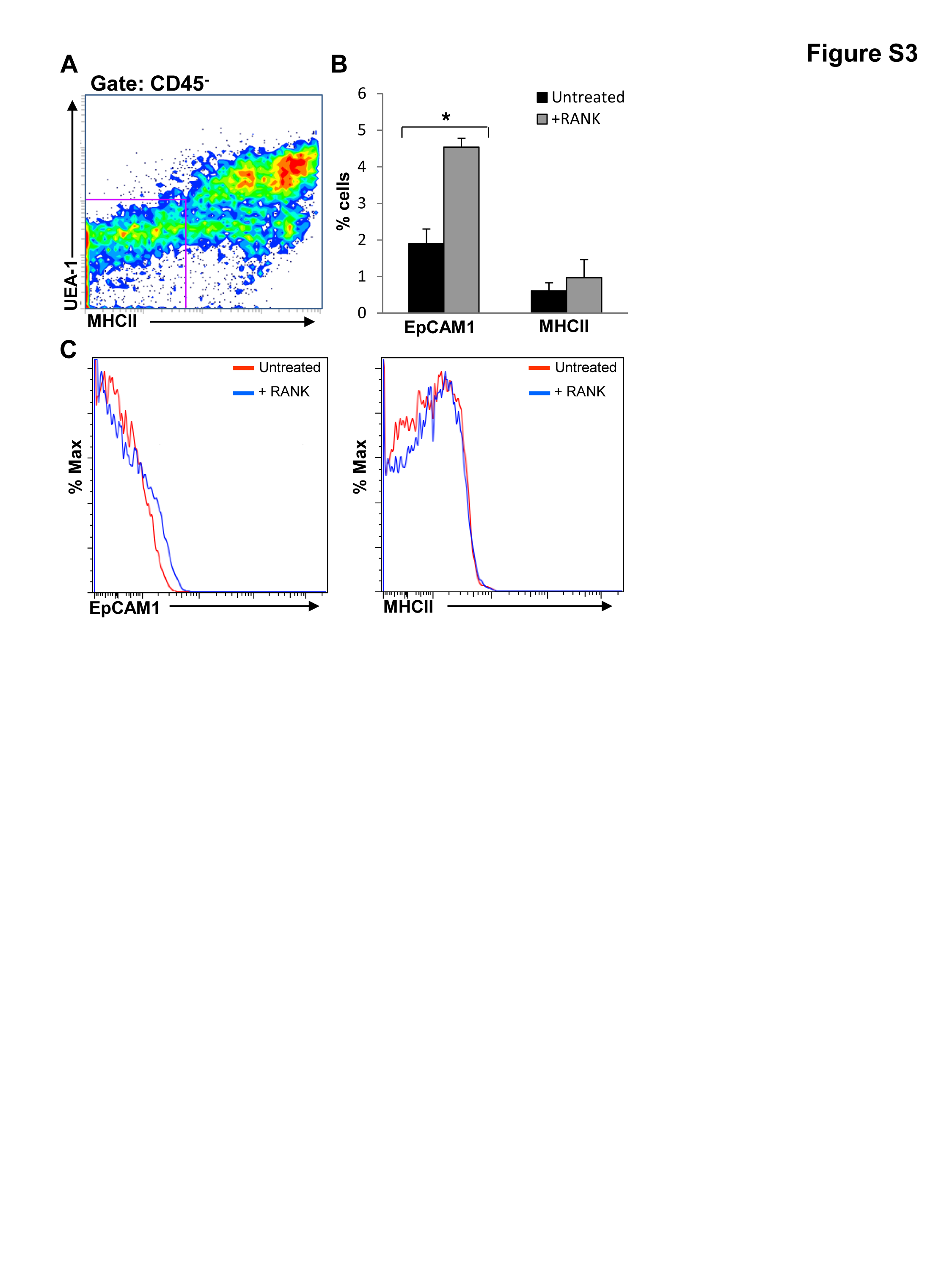

Supplement: Figure S3 — In vitro stimulation of P1–P4 cells with anti-RANKL antibody leads to expansion of EpCAM+ TECs. (A) TEC suspensions were stained with anti-CD45, -MHCII and UEA-1 and cells were sorted based on negative and low UEA-1 binding and negative MHCII expression as shown (rectangle). Six 3-week old mice were pooled together for sorting. (B) Sorted cells were incubated in vitro with anti-RANK antibody and the percentage of EpCAM1+ and MHCII+ thymic epithelial cells were quantified after 3 days in culture by flow cytometry. (C) Expression of EpCAM1 and MHCII on shorted TECs treated with anti-RANK antibody or left untreated for three days in vitro were analyzed on histograms. Bar graphs represent the mean+SEM. n = 3, results were pooled from three independent experiments *p<0.05. (TIF) [file pone.0086129.s003.tif]

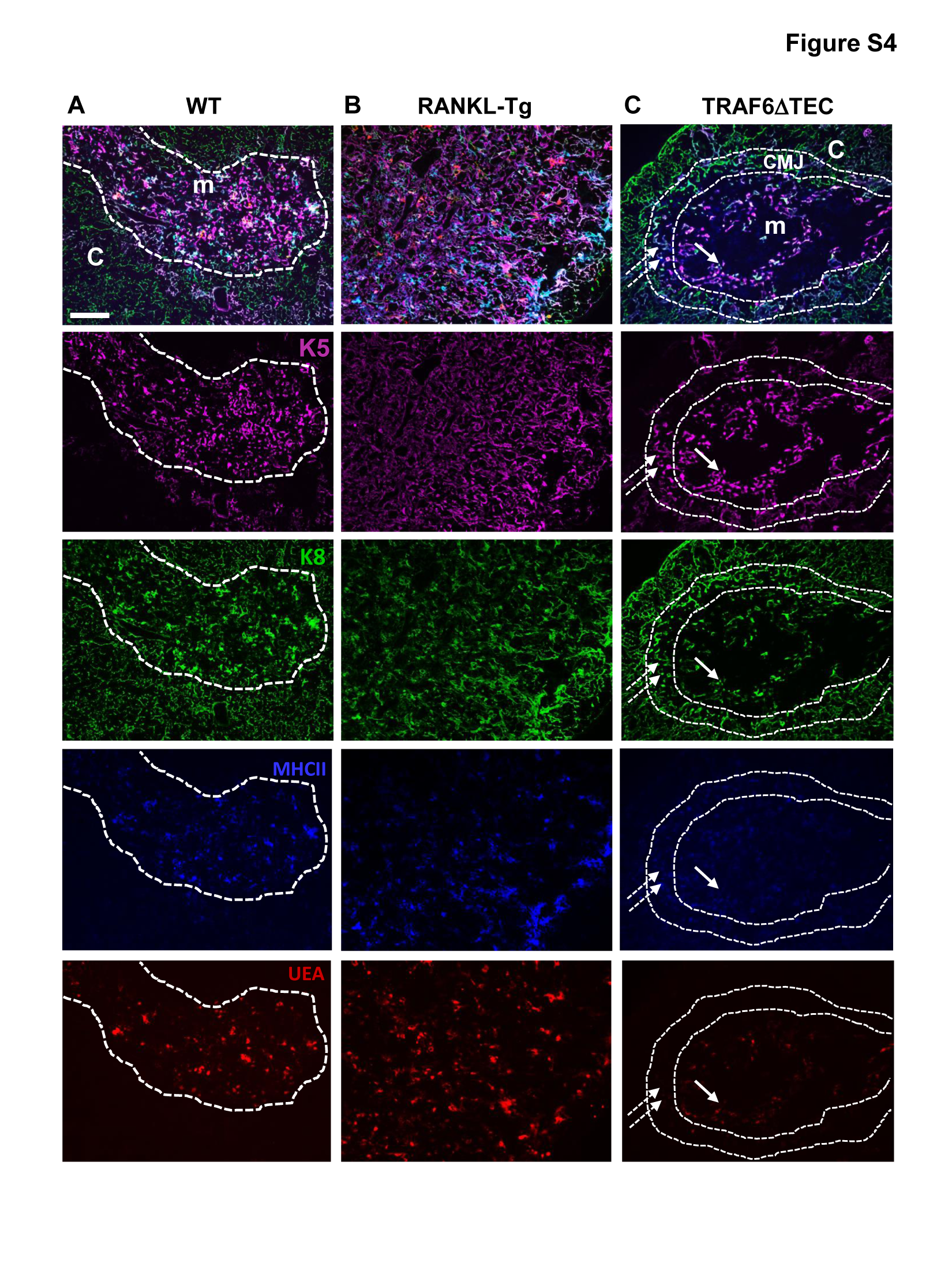

Supplement: Figure S4 — Immature TECs are present in the thymus of Traf6ΔTEC animals. (A–C) Frozen thymic sections from ∼6–8-week old wild type, RANKL-Tg and Traf6ΔTEC were stained with anti-K5, -K8 and -MHCII antibodies and rhodamine-conjugated UEA-1 and analyzed by fluorescence microscopy. K8lowK5lowUEA-1lowMHCIIlow mTECs (solid arrows) and K8lowK5lowUEA-1−MHCIIlow minor cTECs (dotted arrows) are present in the thymus of Traf6ΔTEC cKO mice whereas the medulla is devoid of UEAhiMHCIIhi mature mTECs. Micrographs shown are representative of at least three separate experiments. Scale bar = 100 µm. (TIF) [file pone.0086129.s004.tif]

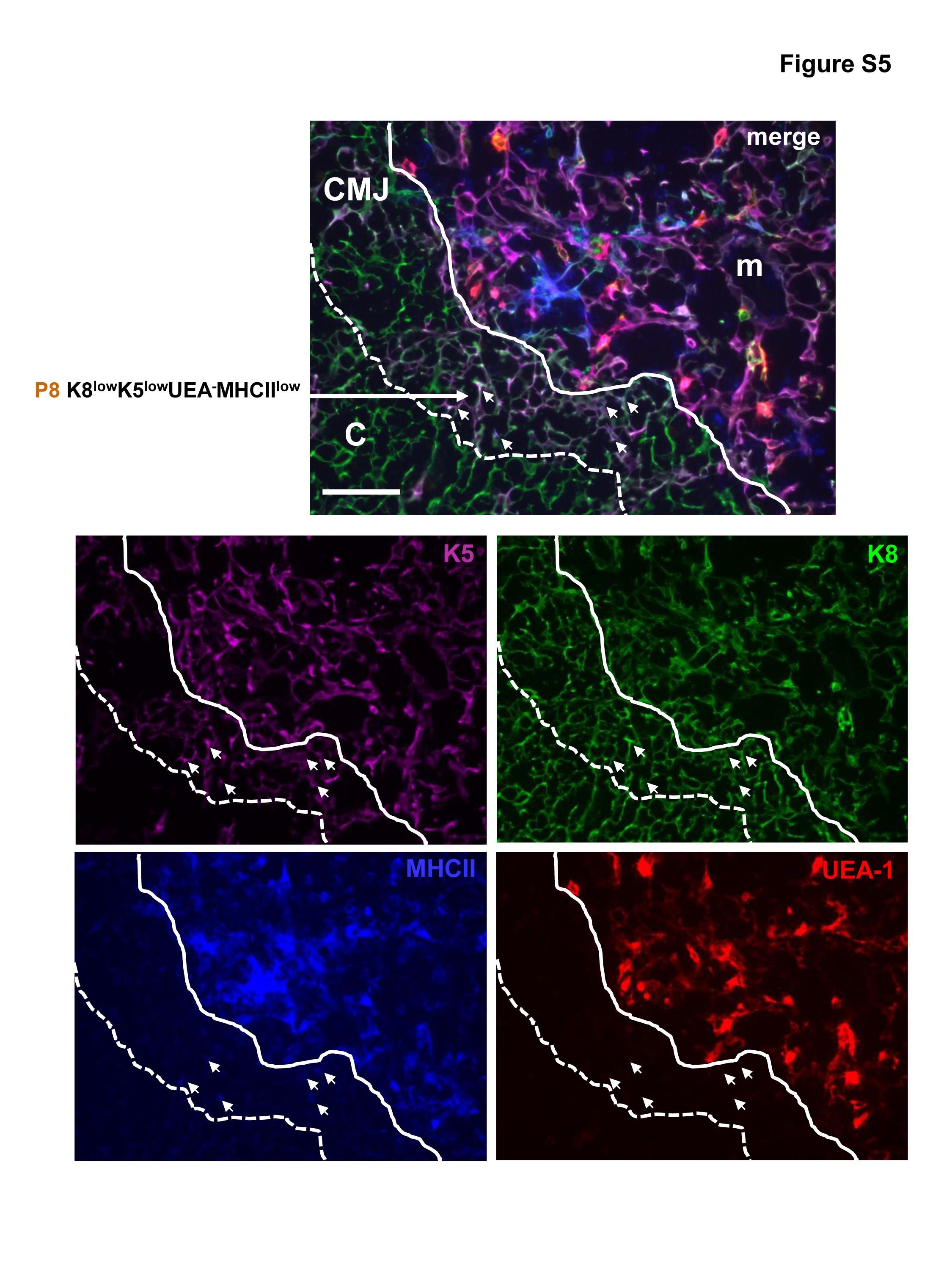

Supplement: Figure S5 — The P8 population is present in the CMJ of the wild type thymus. Frozen thymic sections from ∼6–8-week old wild type mice were stained with anti-K5, -K8, -MHCII antibodies and UEA-1 and analyzed by fluorescence microscopy. Solid and dashed lines demarcate the cortico-medullary junction (CMJ) of the thymus. Arrowheads point to cells that do not bind UEA-1 but express low levels of K5, K8 and MHCII likely representing the P8 population characterized by flow cytometry in Figure 2. Scale bar = 50 µm. (TIF) [file pone.0086129.s005.tif]
